# Targeting Specific Sirtuin Modulation: Emerging Therapeutic Strategies for Healthy Cognitive Ageing and Potential Implications for Alzheimer’s Disease

**DOI:** 10.3390/molecules31193528

**Published:** 2026-10-03

**Authors:** Charlotte McDermott, Michael H. Cole, Madison Pearson, Pia Winberg, Francesca Fernandez

**Affiliations:** 1 School of Behavioural and Health Sciences, Faculty of Health Sciences, Australian Catholic University, Banyo, QLD 4014, Australia; charlotte.mcdermott@myacu.edu.au (C.M.); madison.pearson@myacu.edu.au (M.P.); 2 Healthy Brain and Mind Research Centre, Australian Catholic University, Melbourne, VIC 3065, Australia; 3 Sports Performance, Recovery, Injury and New Technologies (SPRINT) Research Centre, Australian Catholic University, Brisbane, QLD 4014, Australia; 4 School of Medical, Indigenous and Health Science, University of Wollongong, Wollongong, NSW 2522, Australia; pia@venusshellsystems.com.au

**Keywords:** sirtuin, cognition, natural compounds, seaweed, exercise, ageing, calorie restriction, Alzheimer’s disease

## Abstract

Nicotinamide adenine dinucleotide (+)-dependent class III histone deacetylases, widely known as sirtuins (SIRTs), have been found to play key functions in a wide range of biological processes implicated in ageing processes. The downregulation of some specific SIRTs was reported in ageing and in some neurological diseases such as Alzheimer’s disease. SIRT modulator factors, such as diet, exercise and various natural compounds, have recently emerged as a potential new avenue of therapy to modify the progression of Alzheimer’s disease and delay some ageing effects. This review synthesises recent advances in the understanding of the modulatory effects of SIRTs in the context of healthy ageing with a particular focus on cognitive function and implications for Alzheimer’s disease.

## 1. Introduction

As people live longer, the prevalence of age-related diseases has increased, with advanced age being a major risk factor for dementia. Dementia is currently the seventh leading cause of death, with cases projected to rise from 55 million in 2020 to 139 million by 2050 [1,2]. Alzheimer’s disease (AD) is the most common form of dementia, which accounts for at least two-thirds of cases in individuals 65 years and older, and represents a significant burden for our communities worldwide [3]. AD is characterised by progressive cognitive decline, accompanied with multiple pathogenic processes including aberrant amyloid processing and tau hyperphosphorylation, oxidative stress and mitochondrial dysfunction and neuroinflammation [4]. Ageing, one of the strongest risk factors for AD, is also accompanied by progressive chronic neuroinflammation, mitochondrial dysfunction, oxidative stress increase, genomic instability and metabolic dysregulation [5,6,7]. These interconnected processes contribute to the neuronal dysfunction and loss that underlie the damaging effects of ageing and neurological diseases such as AD [5,6,7]. Despite decades of research, current therapies provide limited symptomatic relief with unfortunately no ability to halt or reverse AD progression. Consequently, there is increasing interest in identifying therapeutic strategies that simultaneously target multiple cellular pathways underlying both brain ageing and neurodegeneration [5,8].

Sirtuins (SIRTs), nicotinamide adenine dinucleotide (NAD) (^+^)-dependent class III deacetylases, are conserved protein-modifying enzymes from yeast silent information regulator 2 (Sir2) [9,10]. The founding member, yeast silent information regulator (SIR) 2 (Sir2), was first identified in yeast, and homologous SIRT proteins have subsequently been further characterised in organisms ranging from bacteria to mammals [9,10]. *Sir* genes are nonessential genes that act in trans to mediate transcriptional repression at multiple genomic loci in the budding yeast *Saccharomyces cerevisiae* [11]. Sir2 has been shown to extend lifespan in budding yeast, and more recent studies have reported some anti-ageing effects of SIRTs in additional organisms including worms and flies [12]. Sir2 is closely related to SIRT proteins present in eukaryotic and prokaryotic species [11]. In mammals, seven SIRT isoforms (SIRT1–SIRT7) have been identified, each exhibiting distinct subcellular localisations, substrate specificities and enzymatic activities including deacetylases deacylase and/or ADP-ribosyltransferase activities [5,13]. Through NAD^+^-dependent post-translational modification of histone and non-histone proteins, SIRTs regulate specific cellular processes, such as mitochondrial function and biogenesis, oxidative stress responses, inflammation, deoxyribonucleic acid (DNA) damage repair and genomic stability, autophagy, apoptosis and cell metabolism [5,13]. Since SIRT activity is closely linked to intracellular NAD^+^ availability, the decrease in NAD^+^ availability during ageing may consequently impact the capacity of SIRTs to regulate mitochondrial function, oxidative stress responses, DNA repair and cellular stress adaptation, processes that are particularly important for maintaining neuronal integrity [8]. Increasing evidence suggests that the dysregulation of SIRT pathways contributes to AD pathology, while pharmacological or nutritional activation of specific SIRTs may confer some neuroprotective effects [5,13]. Natural products have become an important source of candidate therapeutics for neurodegenerative diseases due to their ability to target multiple pathological pathways simultaneously. Among these, seaweeds have gained increased attention for their potential use as therapies, particularly in neurodegenerative disorders such as AD [13]. Three primary species of seaweed have been characterised including brown seaweed (*Phaeophyceae*), red seaweed (*Rhodophyta*) and green seaweed (*Chlorophyta*) [14,15]. These seaweeds are rich in bioactive compounds which, for some of them, have shown neuroprotective properties in AD preclinical studies. For example, compounds derived from *Sargassum horneri* have exhibited anti-inflammatory and antioxidant effects in neuronal cells and animal models of AD [16]. Furthermore, polysaccharides and polyphenol compounds extracted from plant and seaweeds, exhibiting anti-inflammatory and antioxidant properties, have been reported to improve memory in AD animal models and, for some of these compounds, to modulate SIRT activity in neuronal cells [16,17,18].

By modulating SIRT activity, key cellular processes maybe influenced, offering potential therapeutic strategies for delaying effects of ageing, such as cognitive decline with potential implications for AD [19,20,21]. This review synthesises briefly the roles of SIRTs in the cell in the context of healthy ageing and AD, including cognitive performance. In addition, endogenous and exogenous factors modulating SIRTs will be discussed, along with their impact in the context of healthy ageing and AD prevention and/or delay of pathological symptoms.

## 2. Overview of SIRTs Involved in Ageing Processes

Seven mammalian homologs of SIRT are distributed across distinct subcellular compartments, enabling them to regulate diverse cellular processes. SIRT1, SIRT2, SIRT6 and SIRT7 are primarily localised to the nucleus, while SIRT3, SIRT4 and SIRT5 are mainly localised in the mitochondria [22,23,24]. SIRT1 and SIRT3 were also found in both nuclear and cytoplasm compartments [22,23,24]. Each SIRT contains a conserved core and a NAD-binding domain, but differ in C- and N-terminal domains [12]. With their distribution across subcellular compartments, SIRTs can regulate a wide range of physiological functions.

### 2.1. SIRT1

SIRT1 was the first SIRT to be identified, sharing the highest homology with Sir2 and the most extensively investigated [25]. One of the main activities of SIRT1 is to deacetylate a variety of key proteins and transcription factors such as forkhead box O (FOXO), leading to the activation of gene expression of antioxidant enzyme superoxide dismutase (SOD) [26,27]. Beyond its NAD^+^-dependent deacetylase activity, SIRT1 also exhibits depropionylase activities, enabling the removal of propionyl groups from lysine residues [28].

SIRT1 plays a significant role in healthy ageing with the promotion of neuronal survival, the enhancement of synaptic plasticity, the regulation of neurodegeneration, and the mediation of adaptive responses to physiological conditions including exercise, fasting and caloric restriction [29,30,31]. Brain-specific SIRT1-overexpressing transgenic mice exhibit significantly extended lifespans in both males and females, with improved phenotypes such as physical fitness and increased expression of the orexin receptor in the hypothalamus, essential in the regulation of sleep patterns and energy balance [32]. Additional studies have also reported the critical role of SIRT1 in multiple cellular processes involved in ageing, such as genomic instability, epigenetic alterations, mitochondrial dysfunction, impaired nutrient sensing, cellular senescence, chronic inflammation and increased oxidative stress [10,27]. By activating peroxisome proliferator-activated receptor gamma coactivator-1α (PGC-1α) and inhibiting nuclear factor kappa-light-chain-enhancer of activated B cells (NF-κB) signalling, SIRT1 promotes mitochondrial biogenesis while suppressing the expression of pro-inflammatory markers, leading to a reduction in inflammation [10,25,31]. Reductions in inflammation and oxidative stress in the cell mediated by SIRT1 was also supported by the deacetylation of FOXO and nuclear factor erythroid 2-related factor 2 (Nrf2) transcription factors in the cell, leading to an improved resistance to cellular damage [5,33]. Beyond regulating transcription factors, SIRT1 also functions as an epigenetic modulator via the deacetylation of histone proteins, leading to chromatin compaction and transcriptional silencing of specific genes, critical in ageing and in sleep regulation [26]. For instance, SIRT1 deacetylates histone proteins and Brain and Muscle ARNT-like Protein 1 (BMAL1) and Period circadian regulator 2 (PER2) factors, both involved in the modulation of the circadian cycle [34,35]. Healthy ageing is accompanied by significant changes in sleep architecture and circadian cycle regulation, resulting in reduced sleep quality and efficiency [36]. Age-related sleep disturbances have significant implications for healthy ageing, as poor sleep is associated with impaired cognitive performance, increased systemic inflammation, metabolic dysfunction and a greater risk of cardiovascular disease and neurodegenerative diseases, including AD [37].

Interestingly, SIRT1 is regulated in a circadian manner and exhibits rhythmic oscillations that coincide with the acetylation of BMAL1 by Circadian Locomotor Output Cycles Kaput (CLOCK), a protein with histone acetyltransferase (HAT) activity, and the acetylation of histone H3 at lysine 9 (H3K3) and lysine 14 (H3K14) [34]. SIRT1 interacts directly with CLOCK and is recruited to the CLOCK/BMAL1 transcriptional complex, modulating chromatin states and the regulation of gene expression of circadian genes [34]. Furthermore, SIRT1 deacetylates PER2, promoting its degradation and contributing to the maintenance of circadian periodicity [35]. As SIRT1 activity is dependent on intracellular NAD^+^ availability, it forms a reciprocal feedback loop with the circadian clock through the regulation of nicotinamide phosphoribosyltransferase (NAMPT), the rate-limiting enzyme of the NAD^+^ pathway. Circadian oscillations in NAMPT expression drive rhythmic NAD^+^ synthesis in the cell, which in turn regulates SIRT1 activity and the activation of circadian gene expression [38]. Consequently, age-associated decline in NAD^+^ availability in cells can reduce SIRT1 activity, contributing to circadian dysfunction, sleep disturbances and metabolic dysregulation observed with age [39]. Furthermore, the genetic ablation of *Sirt1* in a knockout (KO) mouse was shown to lead to the disturbances of the circadian rhythm along with the inability to adjust to a different light–dark cycle, supporting the critical role of SIRT1 in modulating the circadian cycle [40]. More specifically, SIRT1 was reported to contribute to the regulation of slow-wave sleep (non-rapid eye movement (NREM) stage N3), characterised by delta waves [32]. In aged brain-specific SIRT1-overexpression transgenic mice, higher delta power in NREM sleep was reported when compared to aged control mice, suggesting that increased SIRT1 activity may promote deeper and higher-quality sleep [32]. Regulation of these delta waves may occur via the SIRT1/Nk2 homeobox-1/orexin type 2 receptor pathways [32]. Supporting the critical role of SIRT1, targeted *SIRT1* knockdown in lateral hypothalamic nuclei and dorsomedial led to decreased sleep quality in mice [40].

Given that delta-wave activity during NREM sleep is essential for memory consolidation and synaptic homeostasis, the age-related decline in SIRT1 may contribute to the concomitant deterioration in memory, neuronal function and sleep quality observed with ageing [41,42]. While SIRT1 primarily regulates molecular pathways involved in circadian rhythms, synaptic plasticity and sleep homeostasis, SIRT3 complements these functions by maintaining mitochondrial bioenergetics and reducing oxidative stress.

### 2.2. SIRT3

**Figure 1:**
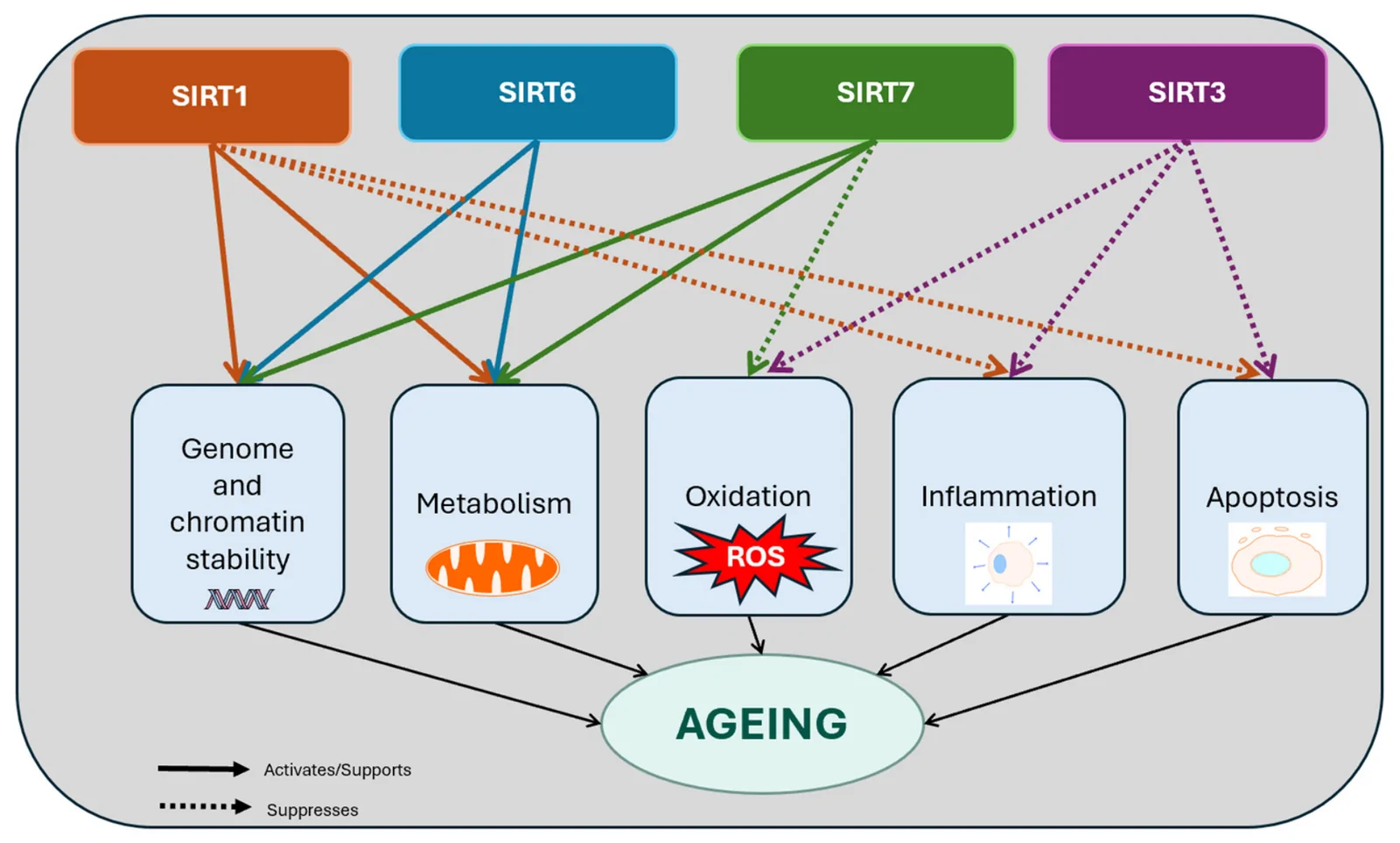
An overview of the SIRTs and their roles across five core biological processes implicated in ageing: genome and chromatin stability, metabolism, oxidative stress (reactive oxygen species (ROS)), inflammation, and apoptosis. The solid arrows indicate the activation/support of a given process, while a dashed line indicates suppression. SIRT1 (orange) and SIRT6 (blue) primarily activate genome/chromatic stability and metabolic pathways while suppressing inflammation and apoptosis. SIRT7 (green) supports genome/chromatin stability, metabolism, and oxidative stress while weakening inflammatory signalling. SIRT3 (purple) promotes metabolic and apoptotic regulation while suppressing oxidative stress and inflammation. Altogether, the dysregulation of these SIRT-mediated pathways converges on the proportion of ageing.

SIRT3 is a mitochondrial NAD^+^-dependent deacetylase with reported decrotonylase enzymatic activity, contributing to antioxidant defence, cell energy metabolism and mitochondrial integrity, all cellular processes in ageing (Figure 1) [5,43,44,45]. SIRT3 substrates include manganese superoxide dismutase (MnSOD) and enzymes involved in the tricarboxylic acid cycle (such as succinate dehydrogenase), fatty acid β-oxidation and oxidative phosphorylation (including Cytochrome c Oxidase) [5,45]. Deacetylation of these enzymes by SIRT3 leads to enhanced mitochondrial adenosine triphosphate (ATP) production and reduced oxidation, essentially to preserve mitochondrial and cell homeostasis [5,26,45,46]. Beyond its established role in regulating mitochondrial bioenergetics and oxidative stress, SIRT3 has also been implicated in maintaining neuronal homeostasis during metabolic stress associated with sleep disruption. Sleep deprivation protocols in mice were found to impair mitochondrial redox homeostasis in locus coeruleus neurons, resulting in increased reactive oxygen species (ROS) accumulation and neuronal damage [47]. These effects were associated with reduced activation of SIRT3-mediated antioxidant pathways, while shorter periods of sleep deprivation induced a compensatory SIRT3-mediated antioxidant response via increased expression of SOD2 and catalase in mitochondria [47,48]. Consequently, age-related declines in SIRT3 activity may increase vulnerability to oxidative stress, impair neuronal maintenance, and contribute to cognitive decline and neurodegenerative processes.

The downregulation of SIRT3 has been reported with ageing in multiple tissues, such as adipose tissue, bone and cardiac muscle, accompanied with loss of mitochondrial efficiency and increased oxidative damage [44,46]. Furthermore, SIRT3 protein expression levels assessed in skeletal muscle were reported to decline with age in sedentary adults, whereas endurance-trained adults exhibited significantly higher SIRT3 expression levels, with no significant age-related difference observed among the trained participants [49]. In addition, *Sirt3* KO mice were shown to exhibit a significantly shorter median lifespan, approximately 19% lower than wild-type control mice, providing evidence that SIRT3 contributes to the maintenance of healthy ageing [50]. SIRT3 is one of the best-characterised members of the SIRT family associated with human longevity [51,52]. Genetic studies have identified genetic polymorphisms within the *SIRT3* gene associated with longevity in elderly individuals [52,53]. For instance, a functional 72 bp variable number tandem repeat polymorphism located in intron 5 of *SIRT3* with enhancer effect on gene reporter was associated with men living over 90 years of age, supporting a potential role of SIRT3 in longevity processes [53].

Decline in mitochondrial capacity has been associated with ageing processes, which also include, at a cellular level, impaired mitochondrial quality control mechanisms and the accumulation of mitochondrial DNA/ribonucleic acid (RNA) damage and/or mutations [5,54]. SIRT3 regulates mitochondrial ATP-dependent protease Lon peptidase 1, mitochondria activity, resulting in the increase in mitochondrial proteostasis and facilitation of the removal of damaged or misfolded proteins [55]. SIRT3 also promotes neuronal resilience through the deacetylation of nicotinamide mononucleotide adenylyltransferase 2, which enhances its interaction with heat shock protein 90 (hsp90), and facilitates the refolding and clearance of aggregated proteins [55]. Consequently, SIRT3-mediated maintenance of mitochondrial proteostasis is increasingly recognised as a potential mechanism for preserving neuronal function and reducing age-related neurodegenerative processes. Maintaining SIRT3 activity, either through direct activation or by preserving NAD^+^ availability, has been shown in experimental models to attenuate cellular senescence and, in some contexts, suppress induction of the senescence-associated secretory phenotype, potentially by preserving mitochondrial proteostasis, redox homeostasis and metabolic function [44]. SIRT3 plays a critical role as a nutrient sensor; when mild metabolic stress occurs, SIRT3 activity is increased in the cell and regulates mitochondria processes [45].

Taken together, the diverse actions of SIRT3 in regulating mitochondrial function, proteostasis, oxidative stress and cellular senescence underscore its central role in maintaining mitochondrial health during ageing (Figure 1). While SIRT3 primarily preserves mitochondrial homeostasis, SIRT6 complements these functions by regulating genomic stability, DNA repair, telomere maintenance and inflammation, highlighting the complementary roles of both SIRTs in promoting healthy ageing.

### 2.3. SIRT6

SIRT6 is predominantly localised in the nucleus, where it functions as an NAD^+^-dependent histone deacetylase, demyristoylase, mono-ADP-ribosyltransferase, long-chain deacylase [56]. Through these enzymatic activities, SIRT6 regulates chromatin remodelling, DNA repair, genome stability cellular metabolism and inflammatory signalling, thereby influencing multiple cellular processes implicated in ageing (Figure 1) [5,57]. Similarly to SIRT3, SIRT6 acts as a nutrient-responsive metabolic sensor, coordinating transcriptional and metabolic adaptations to fluctuations in cellular energy availability [56]. In both male and female *Sirt6*-deficient mice, loss of SIRT6 was associated with severe metabolic defects, including hypoglycaemia, reduced subcutaneous adipose tissue, genomic instability and a marked progeroid phenotype, with most animals dying by approximately 4 weeks of age—findings contributing to evidence of the role of SIRT6 in maintaining cell metabolic homeostasis and health [11]. Conversely, transgenic mice overexpressing *Sirt6* exhibit an extended lifespan compared to wild-type control mice, with effects observed in males but not females, suggesting increased SIRT6 activity may promote longevity under specific physiological conditions [11,58]. Interestingly, female *Sirt6* overexpressing transgenic mice were shown to have lower circulating insulin-like growth factor 1 (IGF-1) and altered phosphorylation of key components of the IGF-1 signalling pathway (including protein kinase Akt and phosphatidylinositol 3-kinase), a pathway implicated in the regulation of lifespan [58]. Beyond its role in metabolic regulation reported in animal studies, SIRT6 also contributes to healthy ageing by maintaining genomic stability and regulating DNA damage responses in cells [5]. Cells deficient in SIRT6 display increased genomic instability, which is partly associated with impaired DNA repair capacity, including defects in genomic base excision repair pathways [56]. Furthermore, loss of both SIRT1 and SIRT6 activity has been shown to promote the accumulation of DNA damage, further highlighting the important roles of these SIRTs in preserving genome integrity during ageing [59]. SIRT6 dynamically associates with chromatin in response to DNA damage, and facilitates the recruitment and stabilisation of DNA repair factors, including DNA-dependent protein kinase catalic subunit at sites of DNA double-strand breaks (DSBs), thereby promoting efficient repair of damaged DNA and maintaining DNA integrity [60]. Considering that the accumulation of DNA damage and genomic instability are recognised hallmarks of ageing, the ability of SIRT6 to maintain DNA repair capacity is considered an important mechanism underlying its protective effects against age-associated cellular decline [61].

Altogether, SIRT6 seems to act as a critical regulator of healthy ageing through its coordinated control of metabolic signalling, chromatin regulation and genome maintenance. By modulating metabolic pathways, preserving DNA repair capacity and maintaining chromatin integrity, SIRT6 contributes to cellular resilience against age-associated stress and genomic instability. However, along with SIRT6, SIRT7, another nuclear SIRT, also plays an important role in chromatin regulation, DNA repair and cellular stress responses, representing another key component of the SIRT pathways involved in longevity and age-related processes (Figure 1).

### 2.4. SIRT7

SIRT7 is the most recently identified member of the SIRT family and is predominantly located within the nucleolus, where it functions as an NAD^+^-dependent chromatin-associated regulator, including maintenance of genome stability and DNA repair [62]. SIRT7 has enzymatic activity including deacetylase and desuccinylase which further contributes to its role within the body [5]. Within the nucleolus, SIRT7 regulates ribosomal RNA (rRNA) transcription by modulating RNA polymerase I activity, thereby facilitating ribosome biogenesis and contributing to cellular growth and metabolic adaptation [63]. In addition, SIRT7 participates in cellular responses to metabolic and oxidative stress, contributing to the maintenance of cellular homeostasis and stress adaptation under challenging conditions such as nutrient deprivation [62,64]. Mechanistically, SIRT7 interacts with chromatin-remodelling complexes and regulates both RNA polymerase I- and RNA polymerase II-dependent transcription, highlighting its role as a broad regulator of gene expression and chromatin states [62,65]. Interestingly, SIRT7 and SIRT6 share interacting proteins, which are involved in chromatin assembly, DNA repair and genomic stability, suggesting synergic effects between these nuclear SIRTs in maintaining cellular and genomic integrity during ageing (Figure 1) [66].

With age, significant reductions in SIRT7 levels were reported in both animal and human tissues [62]. However, increased SIRT7 expression has also been observed in specific tissues, including the frontal cortex of the brain, suggesting that regulation of SIRT7 during ageing may be tissue- and/or cellular-context-dependent and influenced by local physiological demands [62]. Despite these tissue-specific expression patterns, age-related reductions in NAD^+^ availability may limit SIRT7 enzymatic activity and consequently impair its cellular functions, highlighting the importance of NAD^+^ availability in regulating SIRT7 activity in the context of ageing [67]. Consistent with this role, *Sirt7*-deficient mice exhibit premature ageing phenotypes, including degenerative cardiac hypertrophy, impaired stress tolerance and reduced lifespan in a mice study [68]. Furthermore, in a mouse model of Hutchinson–Gilford progeria syndrome, progerin-mediated destabilisation and increased proteasomal degradation of SIRT7 contributed to pathological ageing phenotypes, whereas restoration of SIRT7 expression improved disease characteristics and extended lifespan [62,69]. Loss of SIRT7 activity during ageing has also been associated with increased ROS production and impaired oxidative stress regulation in cells, further linking SIRT7 dysfunction to mitochondrial dysfunction and cellular decline [5]. The impact of ageing on SIRT7 expression and activity appears to be tissue-specific and context-dependent, with alterations of SIRT7 potentially affecting essential cellular functions, including rRNA transcription, ribosome biogenesis and genome stability in response to cellular stress. Therefore, strategies aiming at preserving NAD^+^ availability or maintaining SIRT7 activity may represent potential approaches to support cellular resilience during ageing and age-associated disease such as AD. While SIRT1, SIRT3, SIRT6 and SIRT7 seem to play important roles in pathways implicated in ageing, the remaining SIRTs, SIRT2, SIRT4 and SIRT5, may also contribute to processes associated with ageing and age-related disease.

### 2.5. Other SIRTs: Emerging Evidence for Roles in Ageing

Among the less extensively characterised SIRTs, SIRT2, SIRT4 and SIRT5 have increasingly been implicated in cellular processes associated with ageing. SIRT2 is predominantly localised in the cytoplasm but can shuttle between the cytoplasm and nucleus, where it regulates a range of substrates (including histone proteins) involved in cellular homeostasis, stress responses and genomic stability [70]. Both in vitro and in vivo studies have implicated SIRT2 as a potential regulator of ageing and age-related pathologies [71,72]. However, its precise role remains controversial, with evidence suggesting that SIRT2 may exert either protective or detrimental effects depending on the tissue and cellular context [73,74]. For instance, SIRT2 expression in peripheral tissues appears to decline with ageing, with reduced SIRT2 mRNA and protein levels reported in haematopoietic stem cells and macrophages issued from aged mice, as well as in peripheral blood mononuclear cells from older adults [71]. In contrast, other studies have reported increased SIRT2 expression with age, particularly in CNS where increased levels of SIRT2 protein have been observed in the cortex and spinal cord of mice, and in the hippocampus of senescence-accelerated mice [75,76]. Furthermore, SIRT2 may also contribute to the regulation of inflammatory responses, with potential implications for ageing and neurodegenerative diseases such as AD [77]. However, its precise role remains controversial, as both pro- and anti-inflammatory effects have been reported depending on the cellular, tissue and pathological context [77]. In aged macrophages, reduced SIRT2 expression has been associated with increased acetylation and activation of the NLRP3 inflammasome, whereas SIRT2 overexpression promoted NLRP3 deacetylation and reduced the activation of this inflammasome, supporting a potential anti-inflammatory role of SIRT2 [78]. However, contrasting findings have been reported in LPS-stimulated macrophages and microglial cells, where SIRT2 deficiency or pharmacological inhibition of SIRT2 levels reduced NF-κB activation, nitric oxide production and pro-inflammatory factors [77,79]. Altogether, more work is required to fully understand the molecular mechanisms underlying the role of SIRT2 in each cell type, both in healthy and pathological conditions.

Other emergent studies have shown SIRT4, located in mitochondria, to potentially delay ageing while extending organismal lifespan [80]. SIRT4 functions are considered as a “metabolic brake” due to its inhibition of glutamate dehydrogenase and mitochondrial respiration [5,81]. By linking nutrient status to ageing-relevant stress responses, SIRT4 promotes DNA damage repair, influences inflammatory and apoptotic signalling, and modulates cell metabolism, thereby affecting tissue ageing and cellular senescence [80]. However, the role of SIRT4 in ageing appears to be highly tissue -and context-dependent, with increased expression of SIRT 4 reported in ageing and senescent tissues and with reduced expression in others [80,82]. Considering that SIRT is one of the mitochondrial SIRTs least studied, more work is required to further determine SIRT 4 function and pathways and its dynamic changes in ageing or any age-related diseases.

Like SIRT4, SIRT5 is predominantly localised in the mitochondria and functions as an NAD^+^-dependent protein deacylase, with particularly strong desuccinylase, demalonylase and deglutarylase activities and relatively weak deacetylase activity [81,83,84]. SIRT5 contributes to the maintenance of mitochondrial redox homeostasis via desuccinylation of isocitrate dehydrogenase 2 enhancing its activity and promoting NADPH production, resulting in the reduction in cellular ROS levels [81,85]. Considering that ageing is accompanied by mitochondrial dysfunction and increased oxidative activity, SIRT5 may contribute to ageing processes; however further studies are required.

Altogether, SIRTs emerge as key regulators of age-related processes, including mitochondrial homeostasis, metabolic adaptation, genomic stability and cellular stress responses. However, SIRT activities in cells are largely influenced by changes in cellular NAD^+^ availability and environmental factors throughout the lifespan [86]. Consequently, identifying interventions that modulate SIRT pathways has become a new avenue of research in ageing. Lifestyle and nutritional interventions, such as calorie restriction (CR) and exercise, as well as pharmacological and dietary compounds (see Table 1 and Table 2), have been investigated for their ability to modulate SIRT pathways and potentially promote healthy ageing by restoring cellular homeostasis.

## 3. Overview of Modulating Factors of SIRTs

### 3.1. Calorie Restriction

Calorie restriction (CR), defined as a sustained reduction in energy intake without malnutrition, is one of the most extensively studied nutritional interventions associated with improved health and/or lifespan extension across a broad range of organisms, including yeast, rodents and humans [87]. One contributing molecular mechanism underlying these beneficial effects is the activation of NAD^+^-dependent SIRTs’ pathways, notably regulating mitochondrial function and cell metabolic adaptation [10,22,88]. SIRT1 is particularly responsive to changes in nutrient availability, with its expression and activity influenced by CR in multiple tissues, including the heart, liver, white adipose tissue and brain [10,22,88]. Studies in rodents have demonstrated that long-term CR increases SIRT1 expression in several tissues associated with metabolic regulation and ageing. For example, SIRT1 expression was reported higher in tissues including the kidney, liver and brain in 12-month-old Fischer 344 rats, fed with a 60% CR diet when compared to control rats [89]. Similarly, CR increased hepatic and pancreatic SIRT1 expression in male Sprague–Dawley (SD) rats, accompanied with an improved glucose regulation and lower blood glucose levels compared to controls (Table 1) [90]. However, because improved glucose regulation and reduced blood glucose concentrations are well-established effects of CR, these findings alone do not establish a SIRT1-dependent mechanism [90]. To further explore the cellular mechanisms underlying the effects of CR, Cohen et al. have reported that SIRT1 expression was approximately two fold higher in human embryonic kidney (HEK293T) cells cultured in serum obtained from CR male rats (Fischer 344) compared with cells cultured in serum from *ad libitum* fed rats [89]. Interestingly, a one-year intermittent calorie restriction (ICR) intervention in abdominally obese adults also modulated blood SIRT1 levels, with increases observed in men, whereas in women, SIRT1 responses were associated with BMI-related changes, with greater increases observed among those who experienced less BMI reduction [91]. Altogether, these findings suggest that SIRT1 responses to CR are influenced by sex, tissue type and individual metabolic characteristics.

**Table 1:** A summary of the reported major modulators influencing relevant SIRTs in the context of ageing in preclinical studies.

SIRT Modulators	Chemical Structure	SIRT Changes	Studies	References
CR Or ICR		↑ SIRT1	Animal (Fisher 344 rats with 60% CR) HEK 293 cells with implication of decrease insulin/IGF1 and Bax apoptotic pathways	[89]
			Animal (Male SD rats with 60% CR)	[90]
			Animal (Myocardium of female SD rats with 25% CR)	[88]
			Animal (C57Bl/6 positive female mice (CR or ICR))	[92]
			Animal cell line (SD Cardiomyocytes (CR for 3 weeks))	[93]
		↓ SIRT1	Animal (Myocardium of female SD rats with 45% CR and high-fat diet)	[88]
		↑ SIRT3	Animal (Myocardium of female SD rat with 25% CR)	[88]
			Animal (C57Bl/6 positive female mice brain homogenate after 50 weeks (CR or ICR))	[92]
			Animal (Skeletal muscles tissues of C57BL/6 male mice) with involvement of AMPK/PGC-1α	[94]
			Animal (White and brown adipose tissue of C57Bl/6 male mice 3 months progressing to 60% CR) with the implication of PGC-1α/UCP1 pathways	[95]
			Animal cell line (SD Cardiomyocytes (CR for 3 weeks))	[93]
		↓ SIRT3	Animal (Myocardium of female SD rat with 45% CR and high-fat diet)	[88]
		↑ SIRT6	Animal (C57Bl/6 positive female mice brain homogenate after 50 week (CR or ICR))	[92]
		↑ SIRT7	Animal cell line (SD Cardiomyocytes (CR for 3 weeks))	[93]
Exercise		↑ SIRT1	Animal (Male SD rats doing Moderate-intensity swimming) with involvement of BDNF/CREB pathway	[96]
			Animal (Cardiac and adipose tissue of male Wister rats doing 6 weeks of treadmill running) with implication of SIRT1/FOXO3a pathways	[97]
			Animal (Wistar rats doing treadmill running 60% of VO2max over 6 weeks)	[98]
			Animal (mRNA and mitochondrial Hippocampal neurons in vitro (Sirt3+/+ and Sirt3−/− mice) (voluntary wheel running)) with involvement of glutamatergic and SOD2/MnSOD pathways	[99]
			Animal (FVB/NJ mice doing wheel-caged system training)	[94]
			Animal (WT mice doing endurance exercise training) with implication of AMPK pathways with involvement of AMPK/MnSOD pathways	[100]
		↑ SIRT6	Animal (Trained Sirt6 KO mice doing treadmill running) with implication of CREB/Sox6 pathways	[101]
**Terrestrial-derived** **compounds**				
Resveratrol	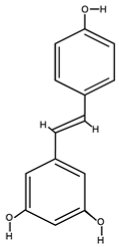	↑ SIRT1	Animal (Inducible SIRT1 KO mice and overexpression SIRT1 mice) Whole Body SIRT1 Overexpressor Mouse) with AMPK pathways	[102]
			Knockdown/rescue experiments, fluorometric SIRT1 activity assay, immunoprecipitation, and pull-down assays with involvement of AMPK/SIRT1 pathway	[103]
			Animal (6 months supplementation and high-fat diet) with AMPK pathways	[102]
Quercetin	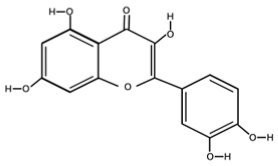	↑ SIRT1	Animal (diabetic-induced atherosclerosis in the carotid artery of rats (2-week supplementation) with involvement of AMPK/SIRT1 pathway	[104]
Fisetin	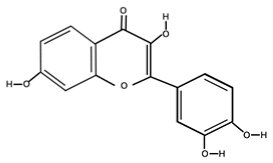	↑ SIRT1	Human osteoarthritis chondrocyte with an involvement of downregulation of NF-κB/IL-1β pathways	[105]
Kaempferol	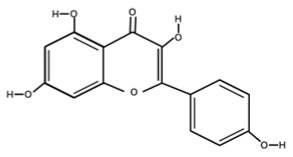	↑ SIRT1	Animal (Lung ischemia–reperfusion injury) With implication of SIRT1-PGC-1α SIRT1/HMGB1/NF-κB pathways	[106,107]
Curcumin	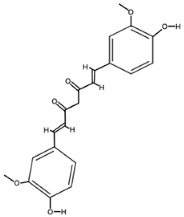	↑ SIRT1	Animal (Female athymic nude xenograft with 200 mg/kg) with involvement of downregulation of NF-κB pathways	[108]
			Cell line (HUVEC with 10 µM) with implication of increase in endothelial Nitric Oxide	[109]
			Animal (Male C57Bl/6 mice with 100 mg/kg/day and 200 mg/kg/day p.o., 5 days) with involvement of downregulation of NF-κB and pyroptosis related proteins	[110]
			Animal (Male SD rats with haemorrhagic shock after lung injury with 50, 200, or 400 mg/kg p.o., 4 days)	[111]
			Cell line (HEC and VSMC with 0.1–1 µM) with involvement of AMPK pathway	[112]
		↑ SIRT1, SIRT3	Animal (Male Wistar rats with 100 mg/kg, oral gavage)	[113]
		↑ SIRT3	Animal (COPD SD rats (100 mg/kg) dissolved in 0.1% carboxy methylcellulose-Na solution) with implication of PGC-1α/SIRT3 signalling pathway	[114]
**Seaweeds**				
Polyphenols *Ecklonia cava*		↑ SIRT1	Animal (obese male C57Bl/6 mice) with implication of AMPK pathways	[115]
Polyphenols *A. nodosum*		↑ Nuclear SIRT1	Cell line (ARPE-19 and WKD cell lines)	[116]
Sterol metabolite fucosterol, (*Ecklonia stolonifera*)		↑ SIRT1	Cell line (3T3-L1 adipocytes) with the involvement of FOXO1 pathways	[117]
Fucoidan	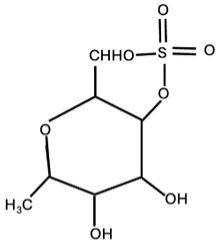	↑ Nuclear SIRT1	Cell line (STZ-treated β cells)	[118]
		↑ SIRT6 No significant changes in SIRT1 and SIRT3	In vitro (biochemical assay vector hSIRT6-pGEX-6P3)	[119]
(*C. nodacaulis*, *F. distichus*, *F. vesiculosus* (*Linnaeus*), *C. tamariscifolia*)		↑ SIRT6	In vitro (biochemical assay vector hSIRT6-pGEX-6P3)	[119]
Fucoidan (*Undaria pinnatifida*)	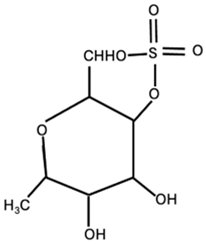	↑ SIRT1	In vitro (cell-based and biochemical assay)	[120]
LMWF (*Saccharina japonica*)		↑ SIRT1	Animal (diabetic *db*/*db* mice) with involvement of SIRT1/AMPK pathways	[121]
LMWF		↑ SIRT3	Animal (Male C57Bl/6 mice with traumatic brain damage)	[122]
Fucoxanthin	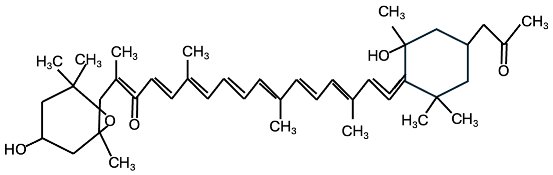	↑ SIRT1	Cell line (Palmitate-induced FL83B cells) Animal (Hepatic and skeletal muscle tissues of diabetic *db*/*db* mice)	[123]
EPHs (*Porphyra* sp., *Caulerpa lentillifera*, *Codium* sp., *Odontella aurita*)		↑ SIRT1	In vitro (biochemical assay)	[124]
EPHs (*Codium decorticatu*, *Bifurcaria bifurcate*)		↓ SIRT1	In vitro (biochemical assay)	[124]
*Chlorella vulgari*		↑ SIRT1	Animal (AlCl3-induced AD rats)	[125]

The arrows indicate how the modulators affect SIRT levels; an up arrow (↑) indicates an increase in the indicated SIRT, whereas a down arrow (↓) indicates a decrease. Abbreviations: CR: Calorie-restricted; ICR: intermittent calorie restriction; SIRT1: Sirtuin one; HEK: human embryonic kidney; IGF1: insulin-like growth factor 1; BAX: Bcl-2-associated X protein; SD: Sprague–Dawley; SIRT3: Sitruin three; AMPK: AMP-activated protein kinase; PGC-1α: peroxisome proliferator-activated receptor gamma coactivator-1α; UCP1: uncoupling protein 1; SIRT6: Sirtuin six; SIRT7: Sirtuin seven; BDNF: brain-derived neurotrophic factor; CREB: AMP-responsive element-binding protein; FOXO3a: forkhead box O3; mRNA: messenger ribonucleic acid; SOD2: superoxide dismutase 2; MnSOD: manganese superoxide dismutase; FVB/NJ: Friend Virus B NIH Jackson; WT: wild type; KO: knockout; SOX6: SRY-box transcription factor 6; HMGB1: High Mobility Group Box 1; NF-κB: nuclear factor kappa-light-chain-enhancer of activated B cells; HUVEC: human umbilical vein endothelial cells; H-EC: human endothelial cells; VSMC: vascular smooth muscle cells; COPD: chronic obstructive pulmonary disease; ARPE-19: immortalised human retinal pigment epithelial cell line; WKD: Wong–Kilbourne Derivative cell line; FOXO1: forkhead box protein O1; STZ: streptozotocin; LMWF: low molecular weight fucoidan; EPHs: enzymatic protein hydrolysates; AlCl3: aluminium chloride.

Beyond SIRT1, CR may also modulate other SIRTs involved in mitochondrial homeostasis and cellular stress responses [5]. In a recent study of female C57/Bl6 mice, SIRT1, three and six expression levels were reported to be elevated in brain homogenates after 50 weeks of CR (or ICR) when compared to baseline levels [92]. These changes were accompanied by reduced evidence of neurodegeneration staining in the frontal cortex and hippocampus [92]. Moreover, in cardiac tissue, a moderate CR diet (25%) increased the protein levels of myocardial SIRT1 and SIRT3 in SD female rats, while a more severe CR (45%) and high-fat diet led to a decrease in the myocardial SIRT1 and SIRT3 protein expression, suggesting that the modulation of SIRT1 and SIRT3 by dietary interventions depend on the degree of energy restriction and more broadly the nutritional context [88]. In skeletal muscle tissue, CR diet significantly elevated SIRT3 levels in muscle when compared to the levels in muscle tissue from ad libitum fed mice, and only 24 h of fasting was necessary to increase SIRT3 levels in the tested tissue [94]. In contrast, prolonged consumption of a high-fat diet and energy-dense diet for three months was associated with reduced SIRT3 levels in skeletal muscle, suggesting that chronic nutrient excess may negatively influence SIRT3 expression and mitochondrial adaptation [94]. Similarly, Shi et al. (2005) reported that CR upregulated SIRT3 mRNA expression in both white and brown adipose tissues of C57Bl/6 male mice following a three-month progressive reduction in food intake, with energy intake gradually reduced to 60% when compared to ad libitum control group mice [95]. In another study examining the short-term effects of CR, three weeks of CR in rats significantly increased the mRNA expression of SIRTs 1–4 and 7 in cardiomyocytes, compared to the levels of mRNA of these SIRTs measured in the cardiomyocytes from the control group fed *ad libitum* (Table 1) [93].

Altogether, these findings suggest that CR modulates SIRT expression and/or activity in a tissue- and context-specific manner, with responses influenced by the degree of energy restriction, nutrient availability and metabolic adaptation. This variability further highlights the complexity of targeting SIRTs as potential mediators of the beneficial effects of CR and as therapeutics targets in ageing. Beyond CR, exercise training has also emerged as an important physiological modulator of SIRT activity partly via its effects on energy metabolism, mitochondrial function and cellular adaptation.

### 3.2. Exercise

Exercise has recently emerged as a promising non-pharmacological strategy for modulating SIRT activity, with evidences suggesting that exercise and CR engage overlapping cellular pathways involved in energy sensing, NAD^+^/NADH ratio and mitochondrial adaptation [126]. Exercise alters cellular energy and redox states including changes in NAD^+^ availability and NAD^+^/NADH ratio, which can influence the activity of NAD^+^-dependent SIRT deacetylases [126]. Increased ATP consumption during exercise leads to the activation of AMP-activated protein kinase (AMPK), which can promote NAD^+^ availability via the NAMPT pathway and facilitate SIRT1 activity [127]. Following its activation, SIRT1 deacetylates its targets such as the (PGC-1α), involved in mitochondria biogenesis processes in skeletal muscle and the forkhead box O1 (FOXO1) and forkhead box O3 (FOXO3) transcription factors [127]. These changes contribute to the transcriptional regulation of genes involved in mitochondrial biogenesis, energy homeostasis, stress and antioxidant defence [127]. Additional animal studies have reported increases in SIRT1 expression following exercise training. As summarised in Table 1, moderate-intensity swimming exercise increased SIRT1 expression of SIRT1 in aged male SD rats, compared to control groups with no exercise [96]. Similarly, six weeks of progressive treadmill training significantly elevated the levels of SIRT1 in cardiac and adipose tissues of aged male Wistar rats [97]. Human intervention studies also provide evidence that regular exercise can modulate SIRT1 expression, although SIRT responses appear to vary according to exercise types, training duration and participant characteristics. For example, twelve weeks of moderate-intensity Pilates exercise performed three times a week significantly increased circulating serum SIRT1 concentrations in overweight women by around 3 fold (*p* < 0.01) [128]. Similarly, an eight-week high-intensity interval training (HIIT) intervention also led to a significant increase in serum SIRT1 levels in postmenopausal women with metabolic syndrome (*p* < 0.05) (Table 2) [129]. Although both interventions reported increases in circulating serum SIRT1 levels, another human study found that a low volume of high-intensity exercise did not significantly alter SIRT1 expression in skeletal muscle levels of healthy young men [103]. These findings suggest that exercise-induced SIRT1 responses may depend on exercise type, duration and examined tissue. Interestingly, six weeks of treadmill exercise with intensity progressively increased to approximately 60% VO_2_ max, significantly increased SIRT1 levels in skeletal muscle tissues, without a corresponding change in circulating SIRT1 levels [63,130]. Consequently, circulating SIRT1 levels may not accurately reflect tissue-specific adaptations occurring in skeletal muscle or other metabolically active tissues when exercising.

**Table 2:** A summary of the reported major modulators influencing relevant SIRTs in the context of ageing in human studies.

SIRT Modulators	Chemical Structure	SIRT Changes	Studies	References
CR Or ICR		↑ SIRT1	Human: mixed sex obese individuals (ICR)	[91]
Exercise		↑ SIRT1	Human: Overweight postmenopausal females (Pilates-turmeric 40–70% intensity)	[128]
			Human: Postmenopausal women with metabolic syndrome (8-week HIIT and physical therapy)	[129]
		No SIRT1 change	Human: Healthy men (HIIT)	[103]
		↑ SIRT3	Human: Endurance-trained individuals (mixed sex) via modulation of NAD^+^/NADH ratio by AMPK	[49]
			Human: Overweight male adolescents (12 weeks moderate-intensity aerobic exercise (treadmill)) with involvement of PGC-1α pathways	[131]
		↑ SIRT6	Human: Chronically exercised individuals (Sourced from public microarray dataset was analysed (GSE9103)	[101]
**Terrestrial** **derived** **compounds**				
Quercetin	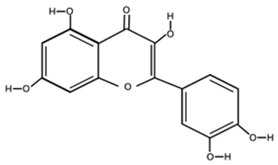	↑ SIRT6	Human: Healthy males (3-week double-blinded, placebo-controlled crossover trial) with SIRT6-Nrf2 axis during acute high-intensity interval exercise	[132]
Curcumin	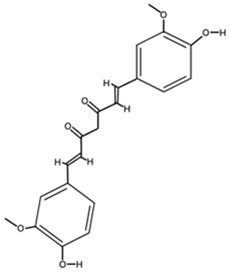	↑ SIRT1	Human (Females with PCOS treated with 1500 mg 3 times/day)	[133]
**Seaweeds**				
*Tetraselmis chuii*		↑ SIRT1	Human (Skeletal muscles) involving NRf2/SIRT1 pathway	[134]

The arrows indicate how the modulators affect SIRT levels; an up arrow (↑) indicates an increase in the indicated SIRT, whereas a down arrow (↓) indicates a decrease. Abbreviations: CR: Calorie-restricted; ICR: intermittent calorie restriction; SIRT1; Sirtuin one; HIIT: high-intensity interval training; SIRT3: Sirtuin three; NAD^+^/NADH: nicotinamide adenine dinucleotide; AMPK: AMP-activated protein kinase; PGC-1α: peroxisome proliferator-activated receptor gamma coactivator-1α; MnSOD: manganese superoxide dismutase; SIRT6: Sirtuin six; Nrf2: nuclear factor erythroid 2-related factor 2; NF-κB: nuclear factor kappa-light-chain-enhancer of activated B cells; IL-1β: interleukin-1 beta; PCOS: polycystic ovary syndrome.

Beyond its effects on SIRT1, exercise also influences SIRT3, which plays a critical role in maintaining mitochondrial function, reducing oxidative stress and preserving cellular homeostasis during ageing processes (see Table 1). Variations in cellular NAD^+^ levels, due to metabolic stress induced by exercise and increased ATP demands, influence the activation of both SIRT1 and SIRT3 [6,126]. As with nutrient restriction, endurance-trained individuals have been reported to exhibit higher skeletal muscle SIRT3 levels than sedentary controls [49]. Furthermore, voluntary wheel running increased SIRT3 mRNA and mitochondrial SIRT3 expression in hippocampal neurons from wild-type mice (*Sirt3*+/+), whereas *Sirt3*-deficient mice (*Sirt3*−/−) were more susceptible to oxidative stress, excitotoxicity and neurodegeneration, demonstrating that SIRT3 contributes to the neuroprotective adaptations induced by exercise [99].

Human intervention reported that twelve weeks of moderate-intensity aerobic exercise increased skeletal muscle SIRT3 levels in overweight adolescents without changes in caloric intake, suggesting that exercise itself can stimulate SIRT3-related mitochondrial adaptations [131]. Similarly, voluntary wheel-running training in mice increased SIRT3 selectively in trained skeletal muscles, whereas no significant change was observed in cardiac tissue, highlighting the tissue-specific nature of this exercise-induced SIRT3 response [94]. Interestingly, female mice exhibited greater SIRT3 levels induced by exercise than males, indicating that sex-specific factors may influence exercise-mediated regulation of SIRT3 in response to exercise [94]. At the molecular level, AMPK appears to be an important upstream regulator of exercise-induced SIRT3 expression. Endurance exercise training increased skeletal muscle SIRT3 protein levels in wild-type (WT) mice, whereas this response was abolished in AMP-activated protein kinase catalytic subunit alpha-2 kinase-dead mice, indicating that intact AMPK signalling is required for exercise-induced upregulation of SIRT3 [100]. However, SIRT3 mRNA expression did not differ between the tested mice genotypes, implying that exercise may regulate SIRT3 only at the protein levels and not for its gene transcription [100].

In contrast, transcriptomic analyses have identified SIRT6 as one of the most strongly upregulated SIRTs at the mRNA levels in skeletal muscle following endurance exercise when compared to sedentary subjects (Table 2) [101]. Increased SIRT6 expression has been accompanied by reduced histone H3 lysine-9 acetylation (H3K9ac) in mice, consistent with enhanced SIRT6 expression and deacetylase activity [101]. Additionally, electrical stimulation of skeletal muscle has been shown to increase SIRT6 protein levels, suggesting that both mechanical muscle contraction and metabolic signalling may contribute to SIRT6 regulation [101]. Considering the role of SIRT6 in DNA repair, genomic stability and glucose metabolism, exercise-induced activation of SIRT6 may represent an additional mechanism through which exercise promotes healthy ageing. However, evidence supporting this pathway remains predominantly preclinical, and further human studies are needed to determine the extent to which exercise-induced changes in SIRT6 contribute to the beneficial effects of exercise on ageing.

Beyond lifestyle-based interventions, such as exercise, dietary-derived compounds have gained more attention for their potential in modulating SIRT activity with resveratrol being one of the most comprehensively studied.

### 3.3. Natural Terrestrial Compounds

#### 3.3.1. Resveratrol

Resveratrol is one of the most studied SIRT-activating natural phenol compounds found in fruits (grapes, berries and tangerine), nuts (peanuts and walnut) and potatoes [135]. Resveratrol has recently been reported to be capable of crossing the blood–brain barrier (BBB) due to its low molecular weight and highly lipid-soluble nature [5]. Resveratrol possesses the ability to activate SIRT1, with response varying according to the levels administered [5]. At lower levels, resveratrol has been reported to enhance SIRT1 activity, which may promote the deacetylation of liver kinase B1 and subsequent activation of AMPK, potentially contributing to an increase in cellular NAD^+^ availability [136]. In contrast, at higher concentrations, resveratrol can impair mitochondrial ATP production by disturbing electron flow through the mitochondrial membrane electron transport chain [137]. Additionally, SIRT1 activation induced by resveratrol may confer anti-inflammatory effects, via the inhibition of NF-κB signalling [5]. In preclinical studies, resveratrol supplementation for six months increases SIRT1 and AMPK activity in mice with a high-fat diet for the remainder of their life, with a lifespan extended for the supplemented group when compared to untreated control group mice [102]. Resveratrol may also activate AMPK via the inhibition of phosphodiesterases, particularly phosphodiesterases-4, leading to the increase in intracellular cyclic adenosine monophosphate (AMP) levels, which can activate downstream signalling pathways, converging on AMPK [13,138]. Subsequent AMPK activation can promote NAD^+^ availability and SIRT1 activity, leading to deacetylation and activation of transcription factors, such as PGC-1α and FOXO regulators, contributing to mitochondrial homeostasis and antioxidant protection [138]. This cascade of molecular events is also proposed to contribute to the neuroprotective and anti-amyloidogenic effects of resveratrol, as supported by emerging evidence from preclinical and human studies [139,140,141]. For instance, in vitro SIRT1 activation by resveratrol in primary rat astrocytes promotes lysosome formation and facilitates amyloid β (Aβ) degradation [140]. In an animal AD model (3X Tg mice), resveratrol supplementation for ten months led to reduced Aβ and p-tau pathology in the hippocampus, with increased AMPK and SIRT1 pathway signalling [139,141]. However, further research is required to elucidate the underlying pathways of resveratrol-induced SIRT1 modulation and associated effects, including clinical trials measuring SIRT1. Although resveratrol remains one of the most studied natural compounds associated with SIRT modulation at the preclinical level, other polyphenolic compounds, including curcumin, have been explored for their potential effects on SIRTs.

#### 3.3.2. Flavonoids

Dietary polyphenols, including quercetin, fisetin and kaempferol, have also demonstrated health benefits via SIRT modulation. Quercetin, a flavonoid abundant in onions, apples and berries, has been reported to modulate the SIRT1/AMPK/signalling pathway and reduce inflammatory markers (NF-κB and interleukin-1 beta (IL-1β) markers) as well as oxidative stress response in diabetic-induced atherosclerosis in the carotid artery of rats when administrated for two weeks [104]. Human studies also reported beneficial effects of quercetin on DNA repair capacity and inflammatory glycomic markers, when administrated for three weeks in healthy male participants from a double-blinded, placebo-controlled crossover trial via the activation of the SIRT6-Nrf2 axis during exercise-induced oxidative stress (acute high-intensity interval exercise) [132]. These findings further emphasise how dietary quercetin may modulate redox-sensitive nuclear and post-translational markers of inflammation in vivo [132].

Along with quercetin, fisetin, another flavonoid compound found in strawberries, apples and persimmons, has received increasing attention due to its senolytic activity and potential ability to regulate longevity-associated pathways [142]. Fisetin was shown to reduce senescence in a subset of cells in murine modelling of a progeroid syndrome and human adipose tissue, demonstrating a cell-type-specific effect [143]. Anti-inflammatory effects were also reported in human osteoarthritis chondrocytes where fisetin administration led to the inhibition of IL-1β-induced inflammation as well as the degradation of SRY-box transcription factor 9, aggrecan and collagen-II potentially through SIRT1 pathway activation [105].

Kaempferol is another natural compound acting on SIRT1 pathways. Kaempferol is a flavonol polyphenol widely distributed in plants, including vegetables and legumes such as cabbage, beans, broccoli, cauliflower and garlic, where it exists primarily in aglycone and glycoside forms [144]. Kaempferol’s beneficial effects on oxidative stress, inflammation and apoptosis were reported in animal studies investigating a model of lung ischemia–reperfusion injury via molecular pathways involving the activation of the mitochondrial SIRT1/PGC-1α pathways and of the SIRT1/HMGB1/NF-κB pathway [106,107].

Although compounds derived from flavonoids possess SIRT modulation activities reported in cells and animal models, further clinical studies are still required to establish their safety, pharmacokinetics, optimal dosing, efficacy and appropriate biomarkers for evaluating their therapeutic potential in humans.

#### 3.3.3. Curcumin

Curcumin, a naturally occurring polyphenolic compound derived from Curcuma longa (turmeric), has emerged as a potential modulator of age-associated pathways due to its antioxidant, anti-inflammatory and metabolic regulatory properties [145]. Similarly to resveratrol, curcumin has been found to modulate cellular pathways such as SIRT pathways. Curcumin administration has been reported to increase SIRT1 expression and activity, increase the NAD^+^ availability and promote anti-inflammatory responses in preclinical studies involving cell lines and animal models [146,147]. For instance, in a preclinical model of head and neck squamous cell carcinoma, curcumin was shown to increase SIRT1 expression and deacetylase activity, accompanied by reduced acetylation of the SIRT1 substrate p53 and inhibition of NF-κB signalling [108].

Extending these findings beyond cancer cell models, curcumin-induced SIRT1 activation has also been reported to attenuate hydrogen peroxide-induced premature senescence in human umbilical vein endothelial cells suggesting a potential protective role of SIRT1 as an antioxidant in the context of stress-induced cellular ageing [109]. Beyond SIRT1, curcumin has also been reported to modulate SIRT3 in preclinical studies. In a rat model of chronic obstructive pulmonary disease (COPD), curcumin increased SIRT3 mRNA and protein expression in skeletal muscle and reduced inflammation and oxidative stress biomarkers (such as IL-6, TNF-α and MnSOD), potentially through activation of the PGC-1α/SIRT3 signalling pathway [114]. Additionally, curcumin administration has been shown to enhance SIRT1 expression in mouse models of acute lung injury [110], and in rat models of haemorrhagic shock after lung injury accompanied by an improved range of pulmonary functions, such as preservation of pulmonary barrier integrity and reduced inflammatory responses [111].

Curcumin’s anti-inflammatory activity, reported in preclinical studies, may be attributed to the inhibition of nitric oxide synthase and cyclooxygenase-2 enzymes via the downregulation of cytokines (tumour necrosis factor alpha (TNFα), interleukins (ILs)) and NF-κB [148]. Through these molecular effects, curcumin may reduce chronic inflammation, a hallmark of ageing, and may improve levels of cell oxidative and metabolic stress. Additionally, curcumin-induced modulations of AMPK and SIRT1 pathways have been associated with improved mitochondrial function and spatial memory performance in mice when compared with control groups [5]. Interestingly, a randomised, double-blinded, placebo-controlled pilot trial has reported an improvement of cognitive performance assessed by Mini-Mental State Examination (MMSE) scoring and a significant decrease in serum inflammatory markers (TNFα and IL-6) after 24 weeks of curcumin–galactomannan complex supplementation [149]. However, this finding must be considered with caution due to the small number of participants (*n* = 16 per group). Further preclinical studies are needed to elucidate the molecular pathways through which curcumin modulates SIRT signalling and to establish whether these mechanisms translate into meaningful physiological benefits. Importantly, findings from preclinical models cannot be assumed to predict clinical efficacy in humans. Further clinical trials are therefore warranted to assess the efficacy and safety of curcumin-based interventions while accounting for its relatively poor oral bioavailability, rapid first pass metabolism, low intestinal absorption and restricted blood–brain barrier penetration [150]. In addition to terrestrial compounds, marine-derived compounds have been uncovered as potential modulators of SIRT pathways, with brown seaweed receiving increasing interest due to its diverse bioactive components.

### 3.4. Natural Compounds Derived from Seaweeds

#### 3.4.1. Brown Seaweed

Marine-derived bioactive compounds, particularly those obtained from brown seaweed, have emerged as potential modulators of SIRT pathways due to their antioxidant, anti-inflammatory and metabolic regulatory properties. Brown seaweeds contain several bioactive compounds, including fucoidan, fucoxanthin and phlorotannins, which have attracted increasing interest in their potential anti-ageing and health-promoting properties [13].

Fucoidan, a sulfated polysaccharide derived from brown seaweeds, has been shown to modulate SIRT signalling pathways, particularly SIRT1, in preclinical models of metabolic stress. In streptozotocin-treated pancreatic β cells, fucoidan increased nuclear SIRT1 protein expression, while pharmacological inhibition of SIRT1 attenuated its protective effects against cellular damage and apoptosis, suggesting fucoidan-mediated cellular responses are at least partially dependent on SIRT1 activation [118]. Consistent with these findings, fucoidan extracted from *Undaria pinnatifida* enhanced SIRT1 expression by approximately 28.8% in in vitro assays, supporting its potential role as a modulator of SIRT pathways [120]. A study in diabetic *db*/*db* mice reported that the administration of low molecular weight fucoidan (LMWF) derived from *Saccharina japonica* increased the phosphorylation of AMPK and restored reduced SIRT1 levels in diabetic mice [121]. Furthermore, elderly male C57Bl/6 mice with traumatic brain damage treated with LMWF showed increased SIRT3 expression in brain tissue [122]. Fucoidan has also been reported to modulate SIRT6 activity, in addition to its effects on SIRT1 signalling [119]. In vitro enzymatic assays demonstrated that fucoidan enhanced SIRT6 deacetylase activity, while no significant changes were observed for SIRT1 or SIRT3 activities at concentrations of 10 or 100 μg/mL, suggesting a degree of selectivity towards SIRT6 activation for LMWF [119]. Furthermore, several brown seaweed extracts, including *Chondrus nodocaulis* increased SIRT6 activity in vitro, with some extracts inducing up to approximately 25-fold enhancement in SIRT6 activity at a concentration of 1 mg/mL (*Fucus distichus*, *Fucus vesiculosus* and *Chondrus tamariscifolia* [119].

Building on the evidence that fucoidan and other brown-seaweed-derived compounds can influence SIRT activity, bioactive constituents from brown seaweeds have been shown to regulate upstream metabolic pathways associated with SIRT activation. Brown seaweeds contain diverse polyphenols, carotenoids and sterol metabolites that may promote healthy ageing through modulation of the AMPK/SIRT1 pathways [115]. For example, polyphenolic extracts from *Ecklonia cava* have been reported to activate AMPK and increase SIRT1 expression in an obese mouse model, suggesting potential effects on metabolic dysfunction associated with ageing and obesity [115]. Similarly, in a cell line of human conjunctival epithelial cells, supplementation with *Ascophyllum nodosum* extract enhanced nuclear SIRT1 activity [116]. As reported in Table 1, the administration of sterol metabolite fucosterol, isolated from *Ecklonia stolonifera*, has also been shown to increase SIRT1 expression and to reduce FOXO1 phosphorylation in 3T3-L1 adipocytes, consistent with enhanced SIRT1-mediated regulation of FOXO1 activity [117]. Furthermore, the treatment of fucoxanthin, a carotenoid abundant in brown seaweeds, also increased SIRT1 expression and AMPK phosphorylation in palmitate-induced FL83B hepatocytes and modulated AMPK signalling in hepatic and skeletal muscle tissues of diabetic *db*/*db* mice [123].

Following investigations of brown seaweed as potential SIRT modulators, studies have also examined green and red seaweeds. These seaweed groups contain diverse bioactive compounds that may modulate SIRT levels with potential effects for ageing.

#### 3.4.2. Additional Types of Seaweeds

Beyond compounds derived from brown algae, other marine bioactive molecules, including algal peptides and sterol metabolites, have demonstrated the potential to regulate SIRT pathways [13].

Treatment with enzymatic protein hydrolysates (EPHs) obtained from several algal red, green and brown algae species have shown SIRT1 modulating activity in in vitro assays [124]. Among these, EPHs from *Porphyra* species, *Caulerpa lentillifera*, *Codium* species, and *Odontella aurita* were associated with SIRT1 activating in vitro capacity, with some extracts producing greater than 80% activation in enzymatic assays [124]. In contrast, hydrolysates derived from the green alga *Codium decorticatum* and the brown alga *Bifurcaria bifurcata* exhibited SIRT1 inhibitory activity [124]. Importantly, hydrolysates derived from *Caulerpa lentillifera* and *Codium* species, correlated with the greatest SIRT1 activation potential, suggesting algal-derived peptides may represent a novel source of SIRT regulators to be tested in the context of ageing in future studies [124].

Compounds derived from seaweeds may also modulate SIRT1 indirectly through activating cellular defence pathways. For example, *Tetraselmis chuii*, a microalga with high SOD activity, increased *SIRT1* transcription in human skeletal muscle via activation of the Nrf2/SIRT1 pathway [134]. Similarly, supplementation with *Chlorella vulgaris* suggested SIRT1 activation in the hippocampus and cerebral cortex in a pharmacological-induced AD rat model, with *Chlorella vulgaris*-loaded niosomes demonstrating enhanced SIRT1 modulation compared to free *Chlorella vulgaris*, potentially due to improved brain delivery and bioavailability [125]. Collectively, these findings expand existing preclinical evidence on the potential of brown-algae-derived compounds in SIRT modulation. A broader range of seaweed-derived bioactive compounds may influence SIRT signalling through indirect mechanisms involving antioxidant defence, metabolic regulation and cellular stress adaptation. Since SIRTs regulate a large range of cellular pathways involved in ageing, the modulation of SIRTs may offer a potential strategy for promoting healthy ageing and mitigating age-related pathologies, such as AD. However, the evidence supporting these effects remains predominantly preclinical, with limited evidence from human studies to date. Further clinical research is therefore required to determine whether the SIRT-modulating effects observed in preclinical models translate into physiological and health benefits in humans.

## 4. SIRT Modulation Strategies in Healthy Ageing and Alzheimer’s Disease

### 4.1. Modulation of SIRT in the Context of Healthy Ageing: Implications for Cognitive and Sleep Functions

Healthy ageing is accompanied by progressive alterations in brain metabolism, mitochondrial function, neuroinflammation, DNA damage and circadian regulation [151], contributing to cognitive decline and disturbances in sleep architecture [152,153]. Considering the critical role of SIRTs in regulating neuronal homeostasis, mitochondrial integrity and circadian rhythms, the modulation of SIRT pathways has emerged as a promising strategy to preserve cognitive function and improve sleep-related outcomes during ageing [153]. Older adults commonly experience reduced sleep duration, decreased slow-wave sleep, increased nocturnal awakenings and altered circadian rhythms, all processes affecting cognitive performance [154]. More specifically, sleep disturbances have been associated with impairments across multiple cognitive domains, including attention, processing speed, memory and executive tasks [152,155]. Human studies examining young and old adults exposed to sleep disruption have consistently showed greater cognitive deficits among older individuals, including poorer performance in word recognition, verbal memory, processing speed, vigilance and executive tasks [154,155,156]. Executive tasks are part of higher-order cognitive processes, which rely heavily on the integrity of the prefrontal cortex [157]. Structural neuroimaging studies have reported that ageing is accompanied with reduction in grey matter volume, cortical thickness and white matter integrity, particularly in the dorsolateral and ventromedial of the prefrontal cortex [158]. Unfortunately, the prefrontal cortex is one of the brain regions most vulnerable to age-related alterations due to its prolonged maturation and high metabolic rate, potentially involving SIRT pathways [158,159]. Strategies modulating SIRT pathways may counteract these processes by maintaining mitochondrial homeostasis, regulating antioxidant responses and supporting brain health [151,152,153,154,155,156,157,158,159].

Among lifestyle-based interventions, physical exercise has been shown to modulate SIRT-mediated signalling pathways, thereby influencing cellular energy homeostasis and enhancing NAD^+^ availability. These adaptations may contribute to the maintenance of mitochondrial function and metabolic homeostasis [160,161]. However, despite some evidence from preclinical and emerging findings in human studies, the extent to which exercise induces the modulation of SIRT signalling remains to be fully understood. This is partly due to the fact that human studies examining the cognitive effects of exercise have not directly assessed SIRT levels and/or activity in relevant human tissues, limiting the ability to establish a mechanistic link between exercise effects and SIRT signalling [162]. For instance, a longitudinal study with healthy older Chinese adults reported that participation in self-selected exercise over one year was associated with significant improvements in overall cognitive performance, particularly for visuospatial function, compared to the non-exercising control group in the study [163]. However, only blood lipidic profiles were assessed in this study, limiting the translation of the results at the molecular levels [163]. Similarly, studies with healthy older adults have reported that higher levels of aerobic fitness are associated with larger hippocampi volumes associated with increased spatial memory performance compared to control participants [164,165]. Although some benefits of exercising were reported, the variation between these studies in exercise types, intensity, duration and participant characteristics limit the direct comparisons between these studies. In addition, some of these studies have largely focused on younger or middle-aged adults, with relatively few studies examining healthy older adults [166]. Although regular exercise is generally associated with the improvement of some specific cognitive domains, such as executive function, processing speed and memory in older adults [167,168], whether these cognitive benefits are mediated via the involvement of SIRT pathways remains to be established [169]. Future human studies integrating well-characterised exercise interventions with direct assessment of SIRT expression and/or activity in relevant tissues, alongside comprehensive cognitive assessments, are therefore required to determine this potential mechanism.

Like exercise, CR induces metabolic adaptations that may increase NAD^+^ availability and potentially modulate SIRT pathways, contributing to improvements in mitochondrial function, oxidative stress resistance and metabolic homeostasis. Consequently, CR has been investigated as a potential strategy to slow aspects of biological ageing, including age-related cognitive decline. The Comprehensive Assessment of Long-term Effects of Reducing Intake of Energy (CALERIE), a phase 2 trial, was a multicentre randomised controlled trial that investigated a prescribed 25% CR in more than 220 healthy, nonobese individuals for two years (NCT00427193). Participants with CR showed improvements in cardiometabolic health, insulin sensitivity and systemic inflammatory markers, but no effects were reported when testing for spatial working memory [170,171,172]. Furthermore, CALERIE participants primarily included young and middle-aged adults (aged approximately 21–50 years), limiting the generalisability of the study findings to older adults and healthy cognitive ageing [170,171,172].

In contrast, supplementation with resveratrol in 23 healthy overweight older individuals over 26 weeks with a pairwise-matched 23 participants receiving a placebo, showed some beneficial effects for cognitive performance in healthy elderly individuals [173]. Indeed, significantly increased word retention over a 30 min delay and Auditory Verbal Learning Test scores (delayed recall and recognition) were observed post-supplementation with resveratrol, when compared with baseline in the tested cohort [173]. However, when investigating the impact of resveratrol supplementation over 26-week trials (NCT01219244) on memory performance in 40 mild cognitive impaired patients aged between 50 and 80 years old, no significant effect was observed for cognition, suggesting different mechanisms for SIRT modulation factors may occur in healthy ageing and pathological conditions [174]. Indeed, cognitive performance may be more difficult to modify once pathological changes associated with mild cognitive impairment have become established in the brain [175]. Individuals with MCI may have already accumulated structural and functional alterations associated with neurodegeneration, including neuronal and synaptic dysfunction, which may make measurable improvements in cognitive performance more difficult to achieve over a relatively short intervention period. Thus, the stage and severity of cognitive decline may represent important factors when considering responses to interventions such as resveratrol. In addition, interindividual variability in resveratrol pharmacokinetics may contribute to inconsistent findings. Resveratrol undergoes extensive intestinal and hepatic metabolism, resulting in low systemic bioavailability and substantial variation in exposure between individuals [176,177]. Genetic polymorphisms in phase II metabolic enzymes involved in the metabolism of natural compounds such as resveratrol, particularly sulfotransferases and UDP-glucuronosyltransferases, may also contribute to this variability [178,179]. Age, gut microbiota composition, dietary factors and concomitant medication use may also influence resveratrol absorption, metabolism and bioavailability [176,180]. However, these factors have rarely been assessed as moderators of cognitive responses in clinical trials.

Furthermore, individual baseline metabolic status may also influence responsiveness to interventions such as CR and resveratrol, given their effects on glucose metabolism, insulin signalling and cellular energy homeostasis [181,182].

Further studies are required to confirm SIRT modulation effects on the preservation of cognitive function during healthy ageing. However, the modulation of SIRTs in cognition has been studied in age-related neurodegenerative diseases such as AD.

### 4.2. Modulation of SIRTs in Age-Related Diseases: Focus on Alzheimer’s Disease

AD is a leading cause of dementia worldwide and is characterised by progressive cognitive decline, behaviour disturbances and memory loss [183]. Despite decades of research, the underlying AD pathogenesis remains unclear. AD hallmarks include amyloid plaques, neurofibrillary tangles (NFTs) along with inflammatory process, astrocytic and microglial activation leading to neuronal and synaptic loss with progression of the disease [184]. Against this backdrop of amyloid and tau-driven neurodegeneration, SIRT1 has emerged as a promising neuroprotective target due to its regulatory influence on both the tau pathology and APP processing reported in preclinical studies. For instance, several studies have suggested that SIRT1 plays an important role in AD pathophysiology, with evidence indicating that its neuroprotective effects may be mediated, in part, through the regulation of neuroinflammation as reported in Aβ25-35-Induced N9 Microglial Cells and N2a-APP695 [185,186]. In animal models of AD, SIRT1 activation has been associated with the reduction in the formation of Aβ plaques and neurofibrillary tangles [187,188]. The underlying mechanisms appear to be a contribution of SIRT1 to a shift in the balance between amyloidogenic and non-amyloidogenic processing of APP, resulting in a reduction in the accumulation of tau and Aβ peptides observed in an AD transgenic mice model [187,189]. Furthermore, SIRT1 reduces amyloidogenic APP processing by inhibiting SIRT2 binding to APP, thereby increasing APP acetylation, promoting APP recycling to the cell surface, and reducing APP retention in endosomal compartments [187,190]. Furthermore, SIRT1 may promote autophagic clearance of Aβ by regulating components of the autophagy machinery, including Atg5, Atg7 and Lc3. In neuronal cell models, the activation of SIRT1 has been associated with increased expression of these autophagy-related proteins and enhanced autophagic degradation of Aβ, although much of the mechanistic evidence is derived from cellular rather than in vivo AD models [190]. SIRT1 may also contribute to the phosphorylation of tau and indirectly to NFT formation, one of the hallmarks of AD. SIRT1 may participate in the leptin-dependent inhibitory effect on tau phosphorylation [191]. This appears to occur via the direct deacetylation of tau at lysine 174 by SIRT1 and its cellular degradation via ubiquitin complex and proteosomes, thereby reducing their aggregation into NFTs [187]. Similarly, SIRT1 deficiency or inhibition in primary neuronal cell culture results in increased tau acetylation and the accumulation of phospho-tau, while enhancing tau deacetylation reduces phospho-tau levels, further supporting that SIRT1-mediated deacetylation may promote tau clearance and limit pathological tau accumulation [192,193]. SIRT1 may also influence tau phosphorylation indirectly via Akt deacetylation inducing its activation, which leads to the inhibition of glycogen synthase kinase-3β (GSK3), decreasing the phosphorylation of tau, one of the substrates of GSK3 [187]. Therefore, the modulation of SIRT activity may influence the accumulation of the NFTs present in neurons, involving the activation of the phospho-tau degradation [192,193]. Furthermore, SIRT1 may contribute to the prevention of axonal degeneration, microglia-mediated Aβ toxicity and polyglutamine toxicity, all processes present in the context of AD [185].

Aside from Aβ and phospho-tau, SIRT pathways also influence molecular processes involved in brain tissue renewal and neuroprotection [194]. The regulation of relevant proteins and genes mediated by SIRTs, for example p53 and NF-kB, may contribute to the potential SIRT neuroprotective effects [185]. In particular, SIRT1-mediated deacetylation of PGC-1 α and transcription factor CREB, an upstream factor of the brain-derived neurotrophic factor (BDNF) pathway, participate in the maintenance of synaptic integrity and neuronal survival [187,188]. In addition, SIRT1 may also attenuate neuroinflammatory signalling by its deacetylation of NF-κB p65 at lysine 310 as reported in preclinical models, leading to a reduced Aβ-induced NF-κB activation in microglia [187]. Additionally, SIRT1 may promote non-amyloidogenic APP processing by upregulating α-secretase A Disintegrin and Metalloproteinase 10 (ADAM 10), leading to the reduction in Aβ production [194].

In addition to its direct effects on molecular pathways, SIRT1’s regulation of histone deacetylation regulates chromatin remodelling and gene expression in AD which may alleviate Aβ-induced transcription dysregulation [187]. SIRT1 has been reported to promote notch signalling by facilitating the release of its notch intracellular domain, leading to an activation transcription of neurogenesis-related genes [194]. Evidence also suggests the notch pathway is essential not only for neurogenesis but as well for the differentiation of newly generated cells in response to pathological injury [194]. Furthermore, studies have reported reductions in both SIRT1 and SIRT3 mRNA/protein expression in AD *postmortem* brains in correlation with the duration and stage of the disease [21,195]. Therefore, while SIRT1 exerts its neuroprotective effects primarily through the nuclear and cytoplasmic pathway, SIRT3 has also gained attention for its potential contribution to AD pathophysiology.

In vitro studies examining both AD mouse model and human AD postmortem tissues suggest a neuroprotective role for SIRT3 in CNS mechanisms dealing with mitochondrial stress, including during AD progression [196]. For instance, an upregulation of SIRT3 mRNA expression was reported following temporal and spatial profiles of Aβ accumulation in temporal cortex AD cases when compared with controls [197]. Furthermore, SIRT3-deficient neurons were reported more vulnerable to apoptosis induced by Aβ, compared to WT neurons, further supporting a potential neuroprotective role of SIRT3 [196]. SIRT3 appears to act via a dual mechanism, involving an upregulation of Aβ-degrading enzymes (neprilysin) and the suppression of the BACE1-mediated amyloidogenic pathway [187]. In primary neuronal cultures from hTau transgenic mice and clinical AD samples, an inverse relationship between SIRT3 levels and both acetylated and total tau was observed [187]. Overexpression of SIRT3 suppressed tau accumulation and acetylation whereas SIRT3 deficiency exacerbated pathological changes, supporting SIRT3 as a potential modulator of tau homeostasis [187]. Alongside its neuroprotective role, SIRT3 also regulates neuroinflammatory and neuronal metabolism. SIRT3 may mitigate neuroinflammation by attenuating microglial activation and NF-κB and NLRP3 inflammasome pathways [187]. Consistent with these findings, Sirt3 KO mice have shown accelerated cognitive decline accompanied by amplified neuroinflammatory responses [187]. Overall, these findings suggest that SIRT3 may support neuronal survival by regulating Aβ and tau levels, and neuroinflammatory signalling in the context of AD. Given the neuroprotective properties of both SIRT1 and SIRT3, research has focused on identifying interventions proficient in enhancing their activity, including exercise and resveratrol.

Preclinical studies show regular physical activity, particularly aerobic exercise, may attenuate memory decline and delay aspects of age-related cognitive impairment in individuals with AD [198]. An a preclinical AD animal model, treadmill exercise increased SIRT1 and PGC-1α levels, which were associated with a reduction in Aβ production within the cortex of these NSE/APPsw-transgenic mice [199]. These changes were accompanied by a shift in APP processing towards the non-amyloidogenic pathway and helped reduce the activity of BACE1 [199]. Additionally, the upregulation of mitochondrial SIRT3 in hippocampal neurons induced by exercise was accompanied by neuronal protection against excitotoxic and metabolic stress [99]. Exercise also significantly increased synaptic connectivity within hippocampal CA1 in APP/PS1 transgenic mice [200], improving cognitive performance and rescuing long-term potentiation in a pharmacological rat model of AD [201], via the reduction Aβ deposition and tau hyperphosphorylation [202]. In human studies, moderate-intensity aerobic exercise also showed some acute beneficial effects for cognitive abilities in mild AD individuals [203]. However, clinical trials involving individuals with AD present additional challenges due to heterogeneity in disease stage; differences in each participant’s capacity to engage in physical activity; and variability in the type, intensity and duration of exercise interventions [5]. Therefore, additional studies are needed to determine the potential therapeutic benefits of exercise in relation to cognition and the modulation of SIRTs in the context of AD.

Resveratrol was reported as another potential SIRT1 modulator, with a potential neuroprotective effect [204]. In an inducible p25 transgenic mouse model of AD, resveratrol supplementation reduced hippocampal neurodegeneration and learning impairment while decreasing acetylation of the SIRT1 substrates PGC-1α and p53 [205]. Chronic resveratrol administration was reported to modulate SIRT1 levels and downstream pathways involved in neurogenesis, including Wnt, NR2E1 and BMP, with associated improvement in cognitive performance in a rodent model [206]. In 3TgXAD mice, supplementation with 100 mg/kg of resveratrol from two months of age to ten months, was associated with enhanced cognition when compared to untreated mice [141]. Furthermore, resveratrol has also been reported to reduce Aβ-related pathology and inflammatory markers in preclinical models; however, evidence for changes in CSF Aβ and plasma inflammatory markers in humans is less consistent [139,141,204]. Resveratrol anti-inflammatory effects have been attributed, at least in part, to the inhibition of NF-κB signalling and to the reduction in activated microglia associated with cortical amyloid plaque formation in a mice model and in vitro cell studies [206]. Although the anti-inflammatory effect of resveratrol was also reported in humans during clinical trials, the absence of direct measures of SIRT activity in many studies limits the ability to establish whether these clinical effects are mediated by SIRTs; therefore, further studies incorporating direct SIRT biomarkers are warranted.

A randomised, placebo-controlled, double-blinded, multi-center 52-week phase two trial with individuals with mild to moderate AD (NCT01504854) investigated the tolerability, safety and treatment effects of resveratrol on AD hallmarks [139]. Participants received 500 mg/day orally initially, with dose escalating by 500 mg increments every 13 weeks to a maximum of 1 000 mg twice daily (NCT01504854). In a subset of participants of this trial resveratrol supplementation was associated with some modulation of neuroinflammation and adaptive immunity responses (including reduced CSF MMP9 and increased IL4 in AD participants) (NCT01504854). Interestingly, resveratrol supplementation was also accompanied with progressive improvements in performing activities of daily living (ADL) scores, in MMSE scores and CSF Aβ40 levels whereas no significant changes in CSF total and/or phospho-tau levels were reported [204]. However, these findings were derived from a relatively small subset of participants (*n* = 38), and larger studies are needed to confirm and validate these observations. Furthermore, SIRT expression or activity was not directly assessed in this clinical trial, limiting the ability to determine whether the observed effects of resveratrol were mediated through SIRT-dependent pathways. Future studies incorporating direct assessments of SIRT expression and activity, alongside cognitive, inflammatory, and AD-related biomarkers, are therefore warranted to clarify the underlying mechanisms of resveratrol’s effects.

Unlike SIRT1 and SIRT3, whose effects are neuroprotective, SIRT2, SIRT4 and SIRT7 appear to contribute to AD pathology under certain circumstances, positioning their inhibition as a potential candidate for a combination therapeutic strategy.

Single-nucleotide polymorphism in the 3’ untranslated region of *SIRT2* was previously associated with AD in a case–control study [207]. SIRT2 has shown to exhibit dual roles in AD with the ability to drive pathological process, in certain circumstances, and exert neuroprotective effects. Interestingly, SIRT2 levels were reported to be increased in male AD patients within the olfactory tract when compared to healthy controls, but this result was not observed in female AD patients [208]. Additionally, a study found an increase in SIRT2 in plasma in AD patients and elderly controls compared to the levels measured in healthy young controls [209]. Interestingly, inhibition of SIRT2 enhances α-tubulin acetylation, reduces tau phosphorylation, strengthens tau-microtubule binding and stabilises microtubules [187]. Streptozotocin (STZ)-treated Tg2576 mice have shown that *Sirt2* overexpression leads to the increase in tau phosphorylation through extracellular signal-regulated kinase (ERK) activation, compared to *Sirt2* knockdown mice which showed attenuated phospho-tau levels [187]. Pharmacological inhibition or genetic knockdown of *Sirt2* has been reported to exhibit neuroprotection in AD animal models, including reductions in neuroinflammation, cognitive decline and amyloid biomarkers, potentially though the attenuation of cytokine production and glial activation [187,210,211,212]. This is supported by a significant positive correlation between CSF phospho-tau and SIRT2 levels among AD patients [213]. Additionally, a study identified SIRT2 being enhanced in a transgenic mouse model with insulin-deficient beta-amyloid precursor protein [214], with the overexpression of Sirt2 inducing tau hyperphosphorylation by ERK activation [214]. SIRT2 has also been implicated with AD, with small-molecule SIRT2 inhibitors (AK-7 and AGK2 previously associated with improved cognition in two transgenic mouse models (3xTg-AD and APP23). These effects may be mediated partially by an alteration of the balance between α- and β-secretase activities, resulting to a reduced Aβ accumulation [215]. More recently, Bai et al. identified that genetic deletion or pharmacological inhibition of SIRT2 improved cognitive performance in APP/PS1 mice, by the increase in APP acetylation promoting the non-amyloidogenic pathway [216]. Therefore, SIRT1 and SIRT2 may be capable of influencing the APP processing in opposing ways [194]. In addition, increased SIRT2’s nuclear activation has been implicated in the epigenetic regulation of inhibiting the expression of Wnt receptors Frizzled (FZD)1 and FZD7 via the deacetylation of H4K16 at their promoters in the hippocampus of humans during early-stage AD and in the hAPP^NLGF/NLGF^ mouse model, contributing to synaptic dysfunction [217]. Interestingly, SIRT2 loss of function using an inhibitor of SIRT2, AK1 in AD cybrid cells or SIRT2 KO restored microtubule stability and improved autophagy, contributing to the elimination of toxic Aβ oligomers and cell survival [187,218]. Another study reported that SIRT2 inhibition with AK7 was associated with increased α-tubulin acetylation and improved cognitive performance in APP/PS1 mice [219]. However, further studies are required to translate preclinical studies investigating the inhibition of SIRT as a potential therapy in AD into potential clinical trials.

Another emerging SIRT potentially involved in AD is SIRT4; however, evidence is limited. Elevated SIRT4 expression correlates with increased Aβ deposition and neuronal apoptosis within APP/PS1 transgenic mouse models [220]. Mechanistically, SIRT4 was shown to promote apoptosis through modulation of the mammalian target of rapamycin (mTOR) pathway and was negatively regulated by the transcription factor signal transducer and activator of transcription 2 (STAT2) in AD-related neuronal injury [220]. Similarly to SIRT4, all these studies are still experimental, and further work is required to further understand the potential role of SIRT in AD.

Beyond SIRT4, SIRT7 has also been implicated in AD-related oxidative stress. For instance, in SH-SY5Y neuronal cells, SIRT7 knockdown attenuated Aβ42-induced ROS production and apoptosis by suppressing the upregulation of NADPH oxidase 4 (NOX4), suggesting that increased SIRT7 may contribute to Aβ-induced oxidative stress [221]. In the same study, SIRT7 mRNA expression was increased in the entorhinal cortex and prefrontal cortex of AD patients compared to controls [221]. In contrast, SIRT7 appears to contribute positively in the maintenance of genome integrity [187]. For instance, a direct role SIRT7 in DSB repair and maintenance of genome integrity via SIRT7-mediated H3K18 deacetylation have been reported using *Sirt7*-deficient cells and mice. Therefore, SIRT7 seems to have opposing effects in the context of AD pathophysiology, potentially contributing to Aβ-induced oxidative damage and neuronal apoptosis while simultaneously and/or independently supporting DNA repair and chromatin stability. Further studies are required to determine whether these effects are dependent on the cellular context and disease stage.

Overall, accumulating evidence suggests that SIRTs may represent a promising therapeutic target for AD, given their involvement in multiple pathological processes, including APP processing, phospho-tau clearance, mitochondrial dysfunction, oxidative stress, neuroinflammation, autophagy, DNA repair, and synaptic function. However, the majority of current evidence supporting SIRT modulation in AD is derived from preclinical studies using cellular and animal models, limiting its consideration as a new avenue of therapy for AD.

## 5. Discussion

In this review, we have discussed how SIRTs regulate multiple molecular pathways, including oxidative stress, chronic inflammation, mitochondrial dysfunction and cellular stress responses, all processes involved in ageing and age-related diseases such as AD [5,10,33,46,86]. Through NAD^+^-dependent deacetylation and acetylation of diverse molecular targets, SIRTs can modulate these pathways and support cell survival [5,10,34,48]. NAD^+^ availability has been proposed to decline with ageing, with depletion closely associated with multiple ageing hallmarks [222]. However, evidence from human studies is still inconsistent and appears to vary according to tested tissues, sex and analytic methodology. A cross-sectional study including 1518 participants over 18 years old, split into five groups according to their respective age range, found no significant difference in whole-blood NAD^+^ levels among these five groups [223]. However, sex-stratified analyses in this cohort identified distinct age-associated patterns, with whole-blood NAD^+^ levels declining with age in men but showing a more variable pattern in women [223]. This suggests the association between ageing and blood NAD^+^ levels are significantly altered by sex. Although there was a trend of NAD^+^ levels declining with age before 50 years, this trend was not significant and disappeared after the age of 50 years [223]. However, since this study is a cross-sectional association between ageing and blood NAD^+^ levels, no temporal relationship or causation could be established [223]. Similarly, a recent study developed and validated a quantitative ultra-high-performance liquid chromatography coupled with high-resolution mass spectrometry (UHPLC -HRMS) method to assess more accurately whole-blood NAD^+^ levels across seven independent human cohorts comprising more than 300 individuals with various ages, levels of activity and diet interventions [224]. Six of these cohorts showed no significant association between whole-blood NAD^+^ levels and age or lifestyle interventions, whereas one cohort of 24 participants supplemented daily with nicotinamide riboside for 5 months showed an expected increase in NAD^+^ levels (positive control) [224]. Altogether, these findings suggest that whole-blood NAD^+^ levels appear to be relatively stable across human ageing. However, these findings should not be interpreted as evidence that NAD^+^ concentrations remain unchanged in all tissues, as circulating whole-blood NAD^+^ may not reflect tissue-specific NAD^+^ availability, including within the brain. For instance, NAD^+^ metabolism was reported disrupted in the brain homogenates of 3xTg AD mice, with significant alterations in metabolites such as nicotinamide mononucleotide (NMN) concentrations in both males and females, with a greater reduction observed in females when compared to WT mice [225]. Further metabolomic analysis reported in this animal model additional significant sex-specific alterations in NAD^+^ metabolites, suggesting that AD-associated disruption of NAD^+^ homeostasis may differ between males and females [225]. Interestingly, amplification of the dysregulation of NAD^+^ was reported in both an AD mouse model (5xFAD) and *postmortem* AD human brains (during the late stage of disease) when compared to controls [226]. Pharmacological restoration of NAD^+^ homeostasis in advanced disease stage in 5xFAD mice resulted in reducing AD biomarkers such as tau phosphorylation and neuroinflammation accompanied with an improved cognitive performance compared to WT mice [226]. Consistent with this finding, a systematic review of preclinical studies reported that NAD^+^ precursors restored brain NAD^+^ levels in rodent models of AD and were associated with improvements in cognitive and neuropathological outcomes in these animal models [227]. Since NAD^+^ is a major co-substrate and co-factor for SIRTs, NAD^+^ metabolism and availability are closely interconnected with SIRT activity, which both appeared impacted in the AD context. Consequently, a combined approach involving the restoration of cellular NAD^+^ availability through nicotinamide riboside supplementation and targeted SIRT modulation may offer synergistic therapeutic benefits. Increasing intracellular NAD^+^ availability may enhance the capacity of SIRTs to regulate cellular processes, while selective modulation of individual SIRTs could help direct these effects towards beneficial pathways. This complementary strategy may therefore enhance the protective effects of SIRT-dependent pathways. However, further investigation is required to determine whether combining NAD^+^ supplementation with selective SIRT modulation can more effectively restore cellular homeostasis and attenuate the multiple pathological processes underlying AD than either intervention alone.

As reported in this review, not all SIRTs exert a cellular protective effect in the context of healthy ageing and pathological conditions. Consequently, selective modulation of SIRTs seems important in order to enhance specifically the protective effects of SIRT-dependent pathways while minimising potentially detrimental effects associated with other SIRT activity. Pharmacological modulation of SIRTs has attracted considerable interest over the past two decades, both as a therapeutic strategy for diseases such as cancer and as a potential approach to targeting age-related processes and promoting healthy ageing [189].

Among synthetic SIRT-activating compounds (STACs), the SIRT1 activator SRT2104 has progressed to early-phase clinical assessment, with a randomised, placebo-controlled phase I trial in healthy older adults aged 60–80 years [228]. Following 28 days of oral administration, SRT2104 was generally well tolerated, with no serious adverse events or dose-limiting toxicities reported in the tested cohort of elderly [228]. Treatment was associated with significant reductions in serum lipids, while reductions in post-exercise phosphocreatine recovery were observed only as a trend, the latter suggesting a potential effect on mitochondrial oxidative phosphorylation [228]. Nevertheless, the small sample size, short treatment duration and exploratory nature of this study preclude conclusions regarding the long-term safety or efficacy of SRT2104 in healthy ageing. Larger and longer-term clinical studies are therefore required to determine whether sustained modulation of SIRT1 by SRT2104 is safe and provides beneficial effects in the context of ageing.

More recent drug discovery research has expanded beyond SIRT1, with the development of more selective activators targeting SIRT3 and SIRT6 [229,230]. Although these SIRT activators have demonstrated some biological activity in preclinical studies relevant to neuroinflammation, oxidative stress and cellular stress responses, the evaluation of these activators in clinical trials remains to be performed [189,229,230]. Further research is therefore required to optimise the potency, pharmacokinetic properties and safety of these synthetic SIRT modulators before clinical translation can be considered.

While synthetic SIRT modulators offer the potential for greater isoform selectivity and pharmacological control, non-pharmacological strategies for modulating SIRT signalling have also attracted great interest, particularly because they may offer a more physiological approach with potentially fewer adverse effects and greater suitability for long-term interventions.

Evidence that CR, exercise and supplementation with naturally derived compounds can modulate SIRT activity in preclinical studies supports the need for further development and evaluation of SIRTs in human interventions in ageing and age-related diseases. Of these interventions, CR and resveratrol supplementation remain the most extensively studied with a growing body of preclinical and early clinical data supporting their ability to modulate SIRTs [70,81,231]. Unfortunately, there are currently limited clinical trials investigating modulating factors of SIRTs, and these human trials are constrained by small subgroup sizes for participants and potential confounding variables affecting the results [2,49,103,129,131,133,139,149,203,204,232]. Therefore, more research is required using SIRT biomarkers to investigate the direct link between clinical benefits and the modulation of SIRT levels particularly in the context of healthy ageing and age-related disease.

Although CR and exercise interventions effectively increased SIRT levels in preclinical studies, translating these findings into clinical trials may be more challenging in older adults and particularly in individuals with AD [233]. Maintaining a low-calorie diet over a prolonged period in a more vulnerable population may increase the risk of malnutrition, which can further impair cognitive and physical capacities [233]. Similar to CR clinical trials, implementing exercise training in a vulnerable cohort can be challenging, particularly among individuals who may experience mobility impairments and balance problems [234]. Indeed, implementation of sustained exercise interventions may become increasingly challenging as cognitive impairment progresses in individuals with AD, with difficulties in maintaining adherence to physical programmes [235]. In addition, the optimal exercise protocols required to modulate SIRT expression or activity remain unclear, particularly with respect to exercise type, intensity and duration, and whether these parameters can also induce changes in SIRT signalling within brain tissue remain to be established.

Consequently, supplementation with natural SIRT modulators may be a more appropriate avenue when moving to human studies in older and AD participants. However, major constraints of these natural compounds include their low bioavailability and challenges associated with crossing the BBB to act on target pathways within the brain. Curcumin has a poor pharmacokinetic profile, characterised by poor absorption, rapid metabolism and elimination, which may limit its therapeutic potential when translated into clinical studies [236]. Although resveratrol showed relatively high oral absorption, its bioavailability range is low (<1%) due to rapid metabolism within the liver and intestine [232]. Compounds derived from seaweeds, including polyphenols, also exhibit low systemic bioavailability as they undergo extensive first pass metabolism [17]. Furthermore, fucoidan’s high molecular weight and polarity may hinder its intestinal absorption, and its large size poses an obstacle to crossing the BBB [17]. Sulfated polysaccharides, such as fucoidan, are generally water soluble; however, their absorption depends on their molecular weight and other structural characteristics [237]. LMWF showed higher absorption and digestion efficiency compared to high-molecular-weight fucoidan; its high viscosity affects its permeability and absorption [237]. An emerging consideration across these studies is the degree and pattern of sulfation as a structure-activity determinant of SIRT modulation. Fucoidan appeared to enhance SIRT6 activity selectively and dose-dependently across species [119]. Fucoidan administration reportedly had little to no effect on SIRT1 or SIRT3, and only a modest reduction in SIRT2 activity when tested in vitro, which may suggest SIRT6 specificity [119]. This selectivity appeared to track more closely with sulfation than with backbone structure, as fucoidans from *F. distichus* and *F. vesiculosus* differ in repeating disaccharide backbone, but both activated SIRT6 similarly [119]. Sulfation and molecular weight have been implicated in fucoidan bioactivity more broadly, with sulfation degree reported to modulate anti-angiogenic activity, and molecular weight of fucoidan has been linked to SIRT3 activation in separate models [119]. However, neither of these parameters were systematically varied against SIRT6 in this study, and their specific contribution to SIRT6 selectivity has not yet been established [119]. This gap reflects a broader challenge within the field, in which fucoidan variability in species compositions, geographic origin and extraction methods may contribute to challenges in reproducibility across in vitro studies within the field. Some degree of standardised structural characterisation alongside bioactivity testing could potentially help clarify structure-selectivity relationships for fucoidan -SIRT interactions. Sulfated polysaccharides from green seaweeds (such as ulvans), despite structural parallels, remain largely unexplored in this context [238]. Given the relative high molecular weight of many sulfated polysaccharides, and their limited intestinal absorption and ability to cross BBB, their effects on SIRT modulation may also be mediated indirectly via gut microbiota fermentation. This process can generate short-chain fatty acids that may activate AMPK-NAMPT signalling and NAD^+^ salvage, potentially limiting inflammation-driven NAD^+^ consumption. Such mechanisms would position sulfated polysaccharides as complementary to direct NAD^+^ precursor supplementation (e.g., nicotinamide mononucleotide) and warrant further studies.

To address absorption and metabolism challenges, new delivery strategies including phytosome and nanoparticles encapsulated with carrier molecules have been recently developed and have shown great potential for increasing the bioavailability of natural compounds in humans [13,239]. Both phytosome and nanoparticle encapsulated delivery have shown improved BBB crossing and have prolonged the systemic half-life of these natural compounds [17]. Phytosome is a phospholipid-based nanocarrier that protects compounds from degradation from the digestive system [240]. The coupling of these natural compounds with negative-charged carbon dot molecules for BBB transportation has shown some promising results in preclinical studies, but remains experimental [241]. In addition, fucoxanthin was shown to have an increased bioavailability when encapsulated with nanoparticles (over 78%) compared to its administration in its nonencapsulated form in an animal model [239], so the prospect of this formulation for application in humans is promising. Additionally, the gel-forming properties for nanogel preparation and the biological versatility of fucoidan offer sustained and biocompatible matrices for controlled drug releases which may enhance BBB and intestinal transportation [242]. These evolving transportation strategies can improve stability and biocompatibility and may improve the therapeutic potential of natural compounds in the future. Despite advancements in nanotechnology, to improve the delivery, solubility and durability of bioactive compounds, the use of these therapeutic avenues requires further investigation.

To progress clinical translation of SIRT modulation interventions, future research should favour dose–response relationships and long intervention durations in ageing populations and at-risk AD populations. Therefore, designed pilot trials are required to evaluate the feasibility, safety and preliminary efficacy of these compounds in increasing SIRT levels and their selectivity for targeting specific SIRTs. In addition, a tailored approach accounting for an individual’s epigenetic and genetic profiles may also be considered in future clinical trials.

## 6. Conclusions

This review highlights the emerging role of SIRTs as key molecular regulators linking metabolism, mitochondrial function, oxidative stress, inflammation, cellular senescence and neurodegenerative processes associated with ageing. Emphasis has been placed on non-pharmacological approaches, including exercise and CR, as well as naturally derived compounds capable of modulating SIRT pathways, including polyphenols, flavonoids and marine-derived bioactive compounds. Preclinical evidence indicates that several of these interventions can enhance SIRT expression or activity and promote beneficial downstream effects, leading to the preservation of neuronal function. However, despite preclinical findings, translation into human ageing populations (and potentially AD patients) remains a major challenge. Much of the current evidence is derived from cellular lines and animal models, which may not fully reflect or translate to the complexity of human ageing, bioavailability issues for natural-derived compounds and tissue-specific responses. Furthermore, variability in compound formulation, dosage, duration of intervention and biological targets has contributed to inconsistent outcomes in clinical studies. Future research should be developed to improve the bioavailability of natural compounds and/or include a combined approach in elderly populations. Given the growing ageing population, the investigation of natural compounds as therapeutic interventions is progressively relevant for both the general population and those experiencing age-related pathological conditions such as AD.

## Data Availability

No new data were created or analyzed in this study. Data sharing is not applicable to this article.

## Abbreviations

The following abbreviations are used in this manuscript:

SIRTs	Sirtuins
AD	Alzheimer’s disease
SIRTs	Sirtuin
NAD	Nicotinamide adenine dinucleotide
Sir2	Silent information regulator 2
HDAC	Histone deacetylase
SIRT	Sirtuin
DNA	Deoxyribonucleic acid
ROS	Reactive oxygen species
FOXO	Forkhead box, sub-group O
SOD	Superoxide dismutase
PGC-1α	Peroxisome proliferator-activated receptor gamma coactivator 1-alpha
NF-κB	Nuclear factor kappa-light-chain-enhancer of activated B cells
Nrf2	Nuclear factor erythroid 2-related factor 2
BMAL1	Brain and Muscle ARNT-Like 1
PER2	Period circadian regulator 2
CLOCK	Circadian Locomotor Output Cycles Kaput
HAT	Histone acetyltransferase
NAMPT	Nicotinamide phosphoribosyltransferase
KO	Knockout
NREM	Non-rapid eye movement
COX	Cyclooxygenase
ATP	Adenosine triphosphate
RNA	Ribonucleic acid
IGF-1	Insulin-like growth factor 1
DSBs	Double-strand breaks
rRNA	Ribosomal RNA
CR	Calorie restriction
HEK	Human embryonic kidney
AL	Ad libitum
ICR	Intermittent calorie restriction
AMPK	AMP-activated protein kinase
FOXO1	Forkhead box protein O1
SD	Sprague–Dawley
HIIT	High-intensity interval training
WT	Wild type
mRNA	Messenger ribonucleic acid
BBB	Blood–brain barrier
Aβ	Amyloid beta
CSF	Cerebrospinal fluid
APP	Amyloid precursor protein
IL-1β	Interleukin-1 beta
HMGB1	High Mobility Group Box 1
COPD	Chronic obstructive pulmonary disease
TNFα	Tumour necrosis factor alpha
ILs	Interleukins
MMSE	Mini-Mental State Examination
LMWF	Low molecular weight fucoidan
EPHs	Enzymatic protein hydrolysates
FVB/NJ	Friend Virus B NIH Jackson
HUVEC	Human umbilical vein endothelial cells
H-EC	Human endothelial cells
VSMC	Vascular smooth muscle cells
PCOS	Polycystic ovary syndrome
STZ	Streptozotocin
NFTs	Neurofibrillary tangles
PP2A	Protein phosphatase 2A
BACE1	β-secretase β-site AβPP-cleaving enzyme 1
ERK	Extracellular signal-regulated kinase
UCP1	Uncoupling protein 1
CREB	cAMP response element-binding protein
